# Activated protein C ameliorates diabetes-induced atherosclerosis by sustaining macrophage efferocytosis

**DOI:** 10.1186/s12933-025-02965-5

**Published:** 2025-10-13

**Authors:** Saira Ambreen, Amna Arif, Saikal Shamkeeva, Ahmed Elwakiel, Surinder Pal, Shihai Jiang, Muhammad Asad Farhan, Zuhir Halloul, John H. Griffin, Berend Isermann, Khurrum Shahzad

**Affiliations:** 1 Institute of Laboratory Medicine, Clinical Chemistry and Molecular Diagnostics, Leipzig University, Paul-List-Straße 13/15, 04103 Leipzig, Germany; 2 Division of Vascular Surgery, Department of General, Abdominal and Vascular Surgery, Otto-Von-Guericke-University, Leipziger Straße 44, 39120 Magdeburg, Germany; 3 Department of Translational Medicine, The Scripps Research Institute, La Jolla, CA 92037 USA

**Keywords:** Atherosclerosis, Diabetes, Macrophage, Efferocytosis, Protein C

## Abstract

Macrophage efferocytosis, essential for the resolution of inflammation and plaque stability in atherosclerosis, is impaired in diabetes. Thrombomodulin (TM) and endothelial protein C receptor (EPCR), key mediators of protein C activation (PC), have vasculoprotective and anti-inflammatory roles, yet their involvement in macrophage efferocytosis in diabetes-induced atherosclerosis remains unclear. Here, we demonstrate that expression of EPCR was reduced in atherosclerotic lesions of diabetic patients compared to non-diabetic controls. In parallel, efferocytosis was impaired in atherosclerotic lesions and in monocytes derived macrophages of diabetic patients. In vitro, treatment with activated PC (aPC) or its cytoprotective selective variant (3K3A-aPC) restored high glucose-impaired macrophage efferocytosis. Mechanistic studies revealed that aPC restored efferocytosis through Arginase-1 and modulation of Rac1-ATF6 signaling. Additionally, macrophage protease-activated receptor 1 (PAR1) was identified as the key receptor mediating aPC’s effects on efferocytosis. Mimicking biased PAR-1 signaling via parmodulin-2 reverses glucose impaired efferocytosis. In vivo, aPC treatment of diabetic ApoE^−/−^ mice increased MerTK expression in atherosclerotic lesions. aPC’s vasculoprotective effects, including the reduction of plaque size, were abrogated upon MerTK inhibition using morpholinos, underscoring the pivotal role of MerTK in mediating aPC’s atheroprotective actions. These findings suggest that impaired TM-PAR1-aPC signaling contributes to defective macrophage efferocytosis in diabetes-associated atherosclerosis and that aPC-based therapies may offer a novel strategy to enhance macrophage function and prevent diabetes induced atherosclerosis.

**Graphical abstract:**

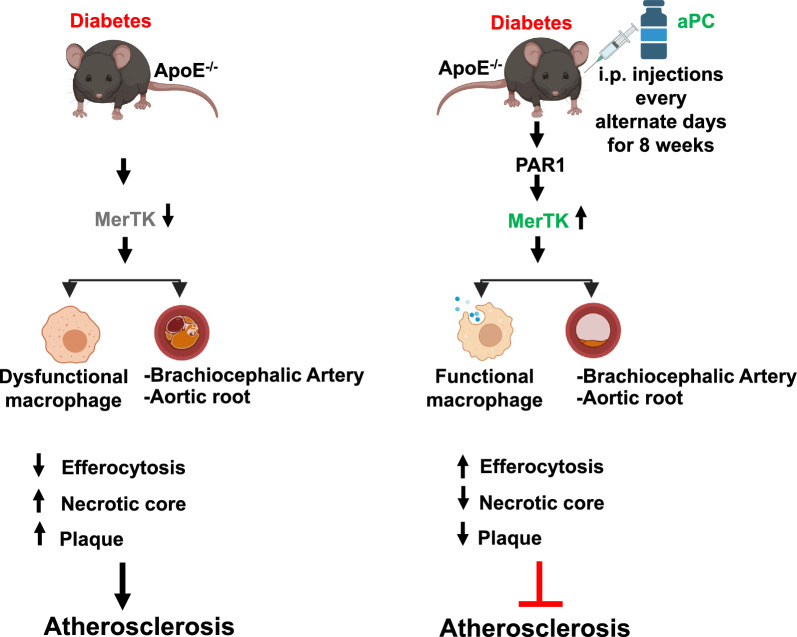

**Supplementary Information:**

The online version contains supplementary material available at 10.1186/s12933-025-02965-5.

## Introduction

Atherosclerosis, a key contributor to cardiovascular disease, is characterized by the accumulation of lipid-laden macrophages, inflammation, and plaque instability [1]. In the context of diabetes, atherosclerotic lesions are often more severe, with increased plaque burden and vulnerability, partly due to impaired resolution of inflammation [1–5]. One critical mechanism for plaque stability and the resolution of inflammation is macrophage efferocytosis, the process by which macrophages clear apoptotic cells [6]. In diabetes, defective efferocytosis leads to secondary necrosis, exacerbating inflammation and promoting plaque instability, which accelerates atherosclerotic progression [5, 7, 8]. The process of efferocytosis is regulated by several key molecules that recognize, engage, and process the cellular material. One such molecule is MerTK (Mer receptor tyrosine kinase), a receptor found on the surface of macrophages that plays a critical role in promoting efferocytosis [6, 9, 10]. Recent studies have shown that MerTK reduction in macrophages contributes to the aggravated atherosclerosis in diabetes-induced atherosclerosis [8]. Despite its importance, the molecular mechanisms underlying impaired efferocytosis in diabetes-induced atherosclerosis remain poorly understood.

Thrombomodulin (TM) and endothelial protein C receptor (EPCR) are key regulators of vascular homeostasis, playing protective roles through their ability to convert protein C to activated protein C (aPC), a potent anti-inflammatory and cytoprotective mediator. While aPC has been shown to provide vasculoprotective effects [11, 12], its role in regulating macrophage efferocytosis in diabetes-associated atherosclerosis has not been explored. Impaired TM-EPCR-aPC signaling has been observed in various vascular diseases, including atherosclerosis [11–15], but its impact on macrophage function, particularly efferocytosis, in the context of diabetes remains unclear.

In this study, we demonstrate that impaired aPC signaling contributes to defective macrophage efferocytosis in diabetes-associated atherosclerosis. The restoration of efferocytosis by aPC was dependent on Arginase-1 (Arg1), a key enzyme in pro-resolving macrophages, and the Rac1-ATF6 signaling pathway, which regulates macrophage efferocytosis. Additionally, protease-activated receptor 1 (PAR1) was identified as a critical receptor through which aPC mediates its effects on efferocytosis. Thus, the aPC-mediated signaling pathway, plays a crucial role in preserving macrophage efferocytosis in diabetes-induced atherosclerosis.

## Methods

See the Supplemental Material for additional information.

### Mice

ApoE^−/−^ (002052) mice were obtained from the Jackson Laboratory (Bar Harbor, ME, USA). C57BL/6 J mice were obtained from Janvier (S.A.S., St. Berthevin Cedex, France). Mice from the same breeding colony were used as controls and the litters were randomly assigned to different experimental groups. Only female mice were used throughout this study in accordance with the approved procedures. All animal experiments were conducted according to standards and procedures approved by the local Animal Care and Use Committee (Landesverwaltungsamt, Leipzig, Germany).

### Atherogenic mouse model

ApoE^−/−^ mice (age 8 weeks) were made diabetic (DM) by injecting streptozotocin (STZ, 60 mg/kg, intraperitoneally, once daily for five consecutive days, freshly dissolved in 0.05 M sterile sodium citrate, pH 4.5), reflecting type 1 DM [11]. Control mice were injected with an equal volume of 0.05 M sodium citrate, pH 4.5, for 5 days. All mice were fed on a normal chow diet. Body weight and blood glucose were measured once weekly. On average, 85–90% of mice became diabetic (blood glucose > 300 mg/dL) within the first 2 weeks, and these were included as diabetic mice in the experiments. Mice not developing persistently elevated blood glucose levels and maintaining blood glucose levels of < 200 mg/dL despite STZ injections were excluded. Hyperglycemia (minimum 300 mg/dL) was maintained for up to 20 weeks. A subgroup of diabetic mice was treated with aPC (1 mg/kg, intraperitoneally, on alternative days) without or with MerTK morpholino (6 mg/kg, intraperitoneally, on alternative days) for 20 weeks after the last STZ injection. For the assessment of plasma amino acid levels in non-diabetes induced atherosclerosis, a subgroup of ApoE^−/−^ mice (age 8 weeks) were fed on high fat diet (HFD) / Western type diet (containing 42% fat, 0.21% cholesterol, 43% carbohydrates, and 15% protein, Ssniff, Germany, catalogue # TD88137) [11, 15–17] for 20 weeks.

### In vivo morpholinos oligomer treatment

Morpholinos (MO) were obtained from Gene Tools, LLC, United States. The following oligonucleotide were used: 5′-TCCGGACAGATGCACGTATCCACCAG-3′ against MerTK (Mus musculus, blocking the translation of transcript variant 1) and 5′- ATCGCCAGG

CGCTTAGCGTAGTGTG-3′ as the random mismatch control. MO were dissolved in PBS and administered intravenously (100 μl; 6 mg/kg, on alternative days) for 20 weeks into a subset of diabetic ApoE^−/−^ mice.

### Analysis of mice

At 28 weeks of age the mice’s body weight was measured and mice were sacrificed [11, 15]. Blood samples were obtained from the inferior vena cava of anticoagulated mice (500 U unfractionated heparin, intraperitoneally). Blood was centrifuged at 2000 × g for 10 min at 4 °C and plasma was stored in − 80 °C. Mice were perfused with ice-cold PBS for 5 min and the heart, aortic arches, including brachiocephalic arteries, were embedded in OCT compound and snap frozen. Upper hearts and brachiocephalic arteries were sectioned at 5 μm thickness.

### Efferocytosis assay

Jurkat cells were collected at a density of 1 × 10^6^/mL in serum-free RPMI-1640 culture medium. Jurkat cells were labelled with Vybrant™ DiO cell labelling solution (V22886, 5 µL/mL of cell suspension in PBS) for 2 min and mixed well by gentle pipetting. Apoptosis was induced by irradiating labelled Jurkat cells under a 254-nm UV lamp for 15 min, followed by an incubation in normal cell culture conditions (37 °C and 5% CO_2_ with saturating humidity) for 2–3 h. The apoptotic cells (ACs) were rinsed once with serum-free RPMI-1640 media and used for experiments. Induction of apoptosis was confirmed by assessing the levels of cleaved caspase-3 (immunoblotting). Bone marrow-derived macrophages were plated in 24-well dishes at a density of 0.18 × 10^6^ cells per well. Labelled ACs were incubated with macrophages (MΦ) for 45 min at a 5:1 (ACs: MΦ) ratio. After 45 min, macrophages were washed three times with PBS to remove unbound ACs, and then the macrophages were fixed with 4% formaldehyde for 20 min, rinsed three times with PBS, and imaged under Keyence microscope.

Alternatively, macrophage efferocytotic capability was determined by flow cytometry (FACS). For the second assay, apoptotic Jurkat cells (AC) were generated as described above. Labelled apoptotic Jurkat cells (1 × 10^6^) were then added to BMDMs at the ratio of 5:1 and co-incubated for 45 min. Then medium was removed, washed with PBS three times and 500 µl of FACS buffer (PBS + 0.5% BSA + 2 mM EDTA) including F4/80 –APC antibody (eBiosceince, 1:2000) was added to the cells. After incubation (30 min), cells were collected and transferred to 5 ml FACS tube and centrifuged at 500 × g for 5 min at 4 °C. Cells supernatant was discarded, and cells were re-suspended in 200 µl of FACS buffer. Cells were then subjected to FACS using APC + FITC filter to determine macrophage efferocytosis efficiency.

### Isolation and culture of peritoneum-derived macrophages

Resident peritoneal macrophages were isolated from mice without elicitation following an established protocol with minor modifications [18]. Mice were euthanized and placed supine on a dissection board. After disinfecting the abdomen with 70% ethanol, the skin was carefully opened to expose the intact peritoneal membrane. Using a 27G needle, 10 ml of RPMI 1640 growth medium was injected into the peritoneal cavity. The abdomen was gently massaged to dislodge adherent cells. A 25G needle attached to a syringe was then used to recover the peritoneal lavage fluid, taking care to avoid organ damage or blood contamination. The cell suspension was immediately centrifuged at 1500 × g for 10 min at 4 °C, washed with PBS and cultured in RPMI 1640 growth medium (supplemented with 30% L929 conditioned media, 20% FBS and 1% penicillin–streptomycin) [19]. Cells were counted, stained with FITC-conjugated F4/80 antibody, and subjected to flow cytometry for the purity test. Additionally, cells were subjected to efferocytosis assay or lysed in RIPA buffer and processed for protein isolation for downstream assays.

### Statistical analysis

The data are summarized as scatter plots with bars. The Shapiro–Wilk test was used to determine whether the data were consistent with a Gaussian distribution. Statistical analyses were performed using unpaired-t test, Mann–Whitney test, Kruskal Wallis test and analysis of variance (ANOVA), and post hoc comparisons were corrected with the Tukey method. Statistics XL (www.statistixl.com) and Prism 9 (www.graphpad.com) software were used for the statistical analyses. Statistical significance was accepted at values of *P* < 0.05.

## Results

### Reduced thrombomodulin and EPCR levels are associated with defective macrophage´s efferocytosis in human diabetes-associated atherosclerotic lesions

**Fig. 1:**
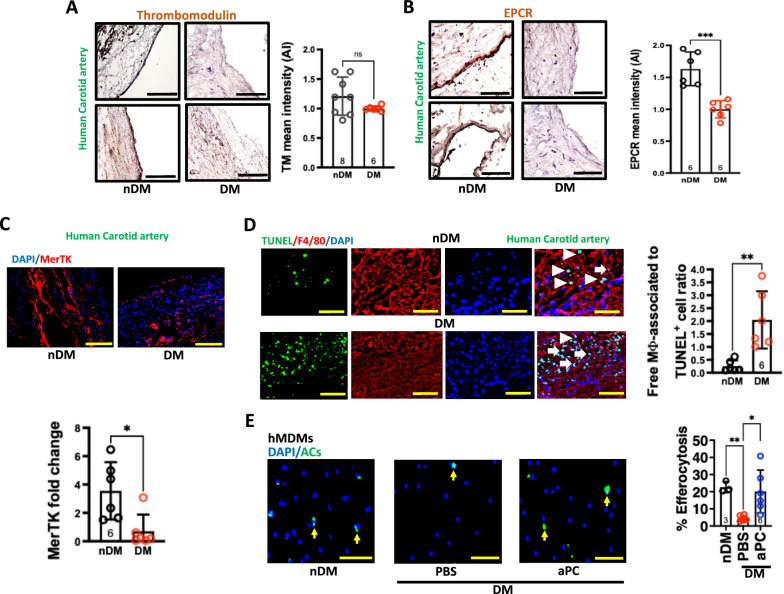
aPC plays a role in enhancing efferocytosis in diabetic human blood-derived macrophages. **A, B** Representative histological images of human carotid arteries showing immunostaining of TM and scatter plot with bars summarising data (**A**). Representative histological images of human carotid arteries showing immunostaining of EPCR and scatter plot with bars summarising data (**B**). nDM (non-diabetic, N = 8), DM (diabetic, N = 6) patients. Scale bar: 200 μm. Data was analysed using unpaired t test. **C** Representative immunofluorescence images showing MerTK expression (red, upper panel), and DAPI nuclear counterstain (blue) and scatter plot with bars summarising data (lower panel) (N = 6). Scale bar: 200 μm. Data was analysed using Mann–Whitney test. **D** Representative images of human carotid artery sections showing macrophages labelled as anti-F4/80 (red) associated with TUNEL positive apoptotic cells (ACs, green), and DAPI nuclear counterstain (blue) (left panel, individual panels, scale bar: 100 μm). The arrow heads indicate the ACs associated with macrophages (merged image). The scatter plot with bars shows quantification of the ratio of free to macrophage-associated ACs (right panel) (N = 6). Data was analysed using Unpaired t test. **E** Representative immunofluorescence images of human peripheral blood mononuclear cells derived macrophages (hMDMs, isolated from non-diabetic (N = 3) and diabetic patients (N = 6)) showing efferocytotic ability of the macrophage (%) to engulf Vybrant DiO labelled apoptotic Jurkat cells (ACs, green) and DAPI nuclear counterstain (blue). Yellow arrows indicate an association of ACs to macrophages (left panel, scale bar: 100 μm). The scatter plot with bars summarising data (right panel). Data was analysed using Welch’s test. PBS, phosphate buffer saline; nDM: non-diabetic; DM: diabetic; aPC: activated protein C. Each dot in the scatter plots represents one biological sample. *P < 0.05, **P < 0.01, ns; non-significant

Given the vasculoprotective roles of thrombomodulin (TM) and endothelial protein C receptor (EPCR), we immunostained cross sections of carotid artery endarterectomy specimens for TM and EPCR from non-diabetic and diabetic patients with atherosclerotic disease. We observed a trend for reduced TM expression within atherosclerotic lesions of diabetic patients as compared to non-diabetic patients (Fig. 1A). Markedly reduced expression levels of EPCR were observed within atherosclerotic lesions of diabetic patients as compared to non-diabetic patients (Fig. 1B).

Macrophage efferocytosis is impaired in diabetes-associated atherosclerosis [20]. Thus, we tested the macrophage efferocytotic ability within atherosclerotic lesion of diabetic and non-diabetic patients. First, we determined macrophage efferocytosis marker, MerTK, in macrophage-rich areas in atherosclerotic lesions. MerTK was lower in diabetic patients (Fig. 1C). Furthermore, impaired efferocytosis was observed in diabetic lesions (Fig. 1D and Supplementary Fig. 1A). Overall, reduced TM and EPCR expression were associated with impaired macrophage efferocytotic ability in diabetes-associated atherosclerosis.

We next determined the efferocytotic ability of peripheral blood human monocyte-derived macrophages (hMDMs) isolated from healthy and diabetic patients ex vivo. hMDMs from diabetic patients showed an impaired efferocytosis (Fig. 1E). Importantly, aPC treatment (20 nM) was sufficient to restore efferocytosis in macrophages isolated from diabetic patients (Fig. 1E).

Collectively, these data suggest that the impaired TM-EPCR-aPC system is associated with defective macrophage efferocytosis in diabetic conditions and that exogenous aPC treatment is sufficient to restore diabetes-impaired efferocytotic ability of macrophages.

### aPC prevents glucose-impaired efferocytotic ability of macrophages in vitro

**Fig. 2:**
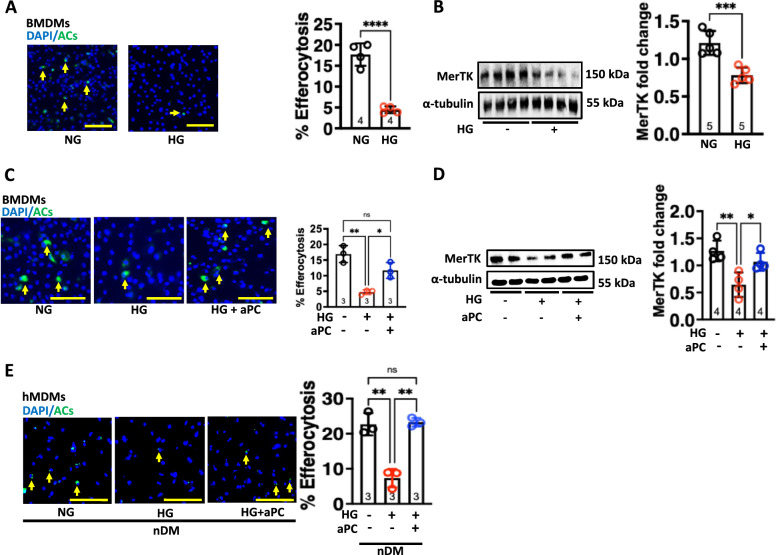
aPC prevents glucose impaired efferocytotic ability of macrophages in vitro. **A** Representative co-immunofluorescence images showing efferocytotic ability of the macrophages (BMDMs, %) to engulf Vybrant DiO labelled apoptotic Jurkat cells (ACs, green) and DAPI nuclear counterstain (blue). Yellow arrows indicate an association of ACs to macrophages (DAPI: blue, nuclear stain, left panel). Scale bar represents 100 µm. Scatter plot with bar graphs summarizing results (right panel) (N = 4). Data was analysed using Unpaired t test. **B** Representative immunoblots showing expression levels of MerTK, α-tubulin: loading control (**B,** left panel) and scatter plot with bar graphs summarizing immunoblotting data for MerTK (**B,** right panel) in BMDMs cells (N = 5). Data was analysed using Unpaired t test. **C** Representative co-immunofluorescence images showing efferocytotic ability of the macrophages (%) to engulf Vybrant DiO labelled apoptotic Jurkat cells (ACs, green) and DAPI nuclear counterstain (blue). Yellow arrows indicate an association of ACs to macrophages (DAPI: blue, nuclear stain, left panel). Scale bar represents 100 µm. Scatter plot with bar graphs summarizing results (right panel) (N = 3). Data was analysed using one way ANOVA with Tukey post hoc comparison. **D** Representative immunoblots showing expression levels of MerTK, α-tubulin: loading control (left panel) and scatter plot with bar graphs summarizing immunoblotting data for MerTK (right panel) in BMDMs cells (N = 4). Data was analysed using one way ANOVA with Tukey post hoc comparison. **E** Representative co-immunofluorescence images showing efferocytotic ability of the hMDMs (%) to engulf Vybrant DiO labelled apoptotic Jurkat cells (ACs, green) and DAPI nuclear counterstain (blue). Yellow arrows indicate an association of ACs to macrophages (**F**, DAPI: blue, nuclear stain, left panel). Scale bar represents 100 µm. Scatter plot with bar graphs summarizing results (right panel) (N = 3). Data was analysed using one way ANOVA with Tukey post hoc comparison. nDM, non-diabetic; NG: normal glucose (5 mM glucose plus 20 mM mannitol), HG: high glucose (25 mM). HG + aPC: HG conditions with additional exposure to aPC (20 nM). Each dot in the scatter plots represents one biological sample. *P < 0.05, **P < 0.01, ****P < 0.0001, ns; non-significant

To assess the effect of glucose on the efferocytotic ability of macrophages, we exposed mouse bone marrow derived macrophages (BMDMs) to either normal glucose (5 mM glucose plus 20 mM mannitol) or high glucose (25 mM, HG) for 24 h. BMDMs were incubated with apoptotic Jurkat cells (ACs, Supplementary Fig. 1B) and efferocytosis efficiency was determined using two approaches, (1) flow cytometry and (2) microscopy (Supplementary Fig. 2 and Fig. 2A, respectively). MerTK was markedly reduced in HG compared to NG exposed macrophages (Fig. 2B).

To evaluate whether pre-treatment with aPC prevents glucose-induced impairment of efferocytosis, BMDMs were pre-treated with aPC for 1 h before exposure to HG. aPC treatment (20 nM) markedly restored efferocytosis (Fig. 2C) and MerTK expression levels (Fig. 2D). Likewise, aPC replenished the glucose-impaired efferocytotic ability of hMDMs (Fig. 2E). Therefore, aPC was able to prevent the HG-mediated impairment of macrophage efferocytosis.

### aPC requires MerTK and Arginase 1 to maintain macrophage efferocytotic ability

**Fig. 3:**
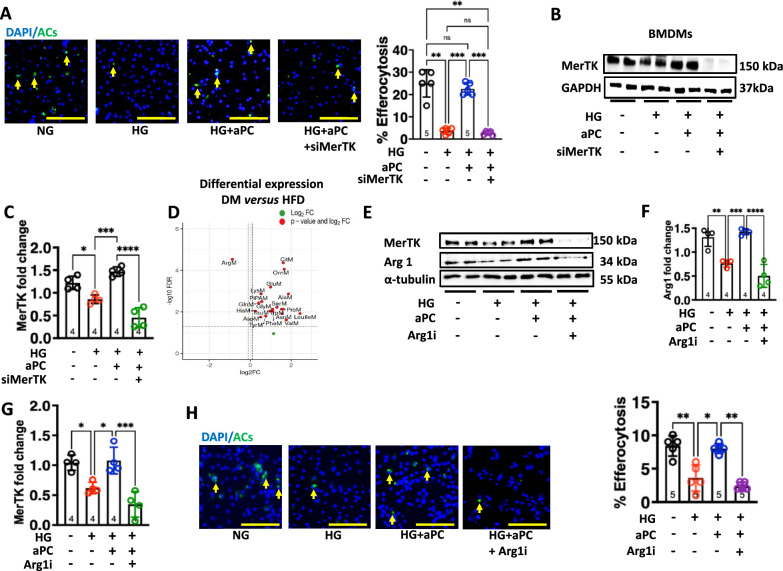
Inhibition of MerTK and Arginase 1 affects the aPC induced efferocytotic ability of the macrophages. **A–C** Representative immunofluorescence images showing efferocytotic ability of the macrophages (%) to engulf Vybrant DiO labelled apoptotic Jurkat cells (**A,** green) after treatment with siRNA targeting MerTK (siMerTK, 5 µM). Yellow arrows indicate an association of ACs to macrophages (**A**, left panel, DAPI: blue, nuclear stain), scale bar represents 100 µm. Scatter plot with bar graphs summarizing results of percentage efferocytosis (right panel). Representative immunoblots showing expression levels of MerTK, GAPDH: loading control (**B**). Scatter plot with bar graphs summarizing immunoblotting data for MerTK (**C**) (**A**: N = 5, **C**: N = 4). Data was analysed using one way ANOVA Tukey post hoc comparison. **D** The volcano plots were generated using amino acid data analyzed by mass spectrometry in plasma from HFD and DM, ApoE^−/−^ mice and setting the threshold for the Log2 (fold change) and − Log10 (FDR) to 2. **E–H** Representative immunoblots showing expression levels of MerTK and Arg1 in BMDMs: α-tubulin, loading control (**E**). Scatter plot with bar graphs summarizing immunoblotting data for Arg1 (**F**) and MerTK (**G**). Representative immunofluorescence images showing efferocytotic ability of the macrophages to engulf Vybrant DiO labelled apoptotic Jurkat cells (**H**, left panel, ACs: green; DAPI: blue, nuclear stain) pre-treated with Nor-NOHA (Arg1 inhibitor, Argi). Scatter plot with bar graphs summarizing results of percentage efferocytosis (right panel) (**F, G**: N = 4, **H**: N = 5). Data was analysed using one way ANOVA with Tukey post hoc comparison (**F, G**) and Kruskal Wallis test (**H**). NG: normal glucose (5 mM glucose plus 20 mM mannitol), HG: high glucose (25 mM). HG + aPC: HG conditions with additional exposure to aPC (20 nM). Each dot in the scatter plots represents one biological sample. *P < 0.05, **P < 0.01, ***P < 0.001, ****P < 0.0001

To determine the functional relevance of MerTK for aPC-mediated efferocytosis maintenance under high glucose conditions, macrophages were transfected with siRNA against MerTK (referred to as MerTK^KD^). While aPC efficiently restored HG impaired efferocytosis in MerTK expressing (Cont) macrophages, it was unable to restore efferocytosis in MerTK^KD^ macrophages (Fig. 3A). aPC failed to induce MerTK in MerTK^KD^ macrophages (Fig. 3B, C). This data suggests that MerTK is necessary for aPC-dependent maintenance of macrophage efferocytosis under high glucose conditions.

To identify the mechanism underlying aPC-mediated efferocytosis, we assessed plasma amino acid levels in diabetic (induced with streptozotocin) or non-diabetic (high fat diet model) ApoE^−/−^ mice by liquid chromatography-mass spectrometry (LC–MS). Alterations in amino acid metabolism are linked with impaired macrophage efferocytosis [6, 10, 21]. Among the studied amino acids, arginine exhibited the greatest reduction in diabetic compared to non-diabetic ApoE^−/−^ mice (Fig. 3D). Arginine is known to promote efferocytosis and resolution of atherosclerosis [6]. Arginine can be metabolized to ornithine by Arginase 1 (Arg1) in pro-resolving macrophages. Decreased expression of Arg1 in macrophages is linked with impaired efferocytotic function [6, 10, 21]. Thus, we studied the role of Arg1. Macrophages exposed to HG conditions showed reduced protein levels of Arg-1 (Fig. 3E, F). Notably, the application of exogenous aPC maintained Arg1 expression (Fig. 3E, F).

To determine the functional relevance of Arg1 for aPC-mediated efferocytosis maintenance under HG conditions, we used a pharmacological approach to inhibit Arg1 with an inhibitor, nor-NOHA (500 mM, named hereon Arg1i) [22]. Of note, aPC was unable to maintain MerTK expression and efferocytosis in macrophages (Fig. 3E–H), indicating that Arg1 is essential for the aPC-dependent increase of MerTK and for the maintenance of macrophage efferocytosis in high glucose conditions.

### aPC engages Rac1-ATF6 signaling axis to maintain efferocytosis in macrophages

To dissect the signalling pathways by which aPC maintains efferocytosis in macrophages under HG conditions, we investigated the involvement of the Rac Family Small GTPase 1 (Rac1) signalling. Previous studies have shown that the process of efferocytosis in macrophages requires Rac1 signalling [6, 23, 24].

**Fig. 4:**
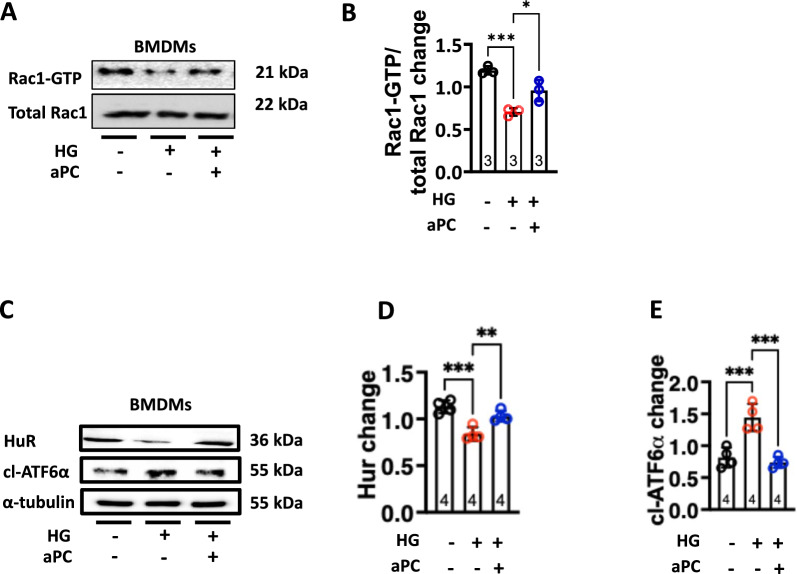
aPC engages the Rac1-ATF6α signaling axis to maintain efferocytosis in macrophages. **A, B** Representative immunoblots showing Rac1-GTP and total Rac1 levels (**A**) and scatter plot with bar graphs summarizing quantification of Rac1-GTP / total Rac1 ratio (**B**), (N = 3). Data was analysed using one way ANOVA with Tukey post hoc comparison. **C–E** Representative immunoblots showing HuR and cl-ATF-6α levels, α-tubulin: loading control (**C**) and scatter plot with bar graphs summarizing immunoblotting data for HuR (**D**) and cl-ATF6α (**E**) (N = 4). Data was analyzed using Kruskal Wallis test (D) and one way ANOVA with Tukey post hoc comparison (E). NG: normal glucose (5 mM glucose plus 20 mM mannitol), HG: high glucose (25 mM). HG + aPC: HG conditions with additional exposure to aPC (20 nM). Each dot represents 1 biological sample.*P < 0.05, **P < 0.01, ***P < 0.001

Importantly, aPC has been shown to regulate Rac1 signalling [25–27]. We determined Rac1 activity in macrophages exposed to HG with or without aPC. Rac1 activity was reduced in macrophages exposed to HG conditions (Fig. 4A, B). Of note, aPC treatment abolished the effect of glucose on Rac1 activation in macrophages (Fig. 4A, B). Rac1 activity in macrophages has been shown to be increased by human antigen R (HuR)—an RNA-binding protein involved in efferocytosis [6]. Accordingly, we determined HuR levels in macrophages exposed to HG with or without aPC. While decreased levels of HuR were observed in HG exposed macrophages, aPC treatment prevented glucose-mediated reduction in HuR (Fig. 4C, D). This suggests that aPC via regulation of HuR-Rac1 signalling maintains macrophage efferocytotic ability under HG conditions.

Maladaptive unfolded protein response (UPR) has been associated with defective efferocytosis and we have previously shown that aPC prevents maladaptive UPR to mediate anti-atherogenic effects by reducing the cleavage of activating transcription factor-6 alpha (ATF6α) under diabetic conditions [28]. To investigate whether aPC prevents cleavage of ATF6α to maintain efferocytosis in macrophages, we determined cleaved (cl)-ATF6α levels. Markedly induced cl-ATF6α levels were observed in HG versus NG exposed macrophages, aPC abolished this induction (Fig. 4C, E). Collectively these data suggest that Rac1-HuR-ATF6 signalling is involved in aPC-mediated maintenance of efferocytosis in macrophages under HG conditions.

### aPC maintains macrophage efferocytotic ability via protease activated receptor 1 (PAR1)

To identify receptors through which aPC restores efferocytosis in macrophages, we determined the expression of protease-activated receptors (PARs) in macrophages. Expression of PAR1, PAR2, PAR3 and PAR4 was readily detectable in BMDMs (Supplementary Fig. 3).

**Fig. 5:**
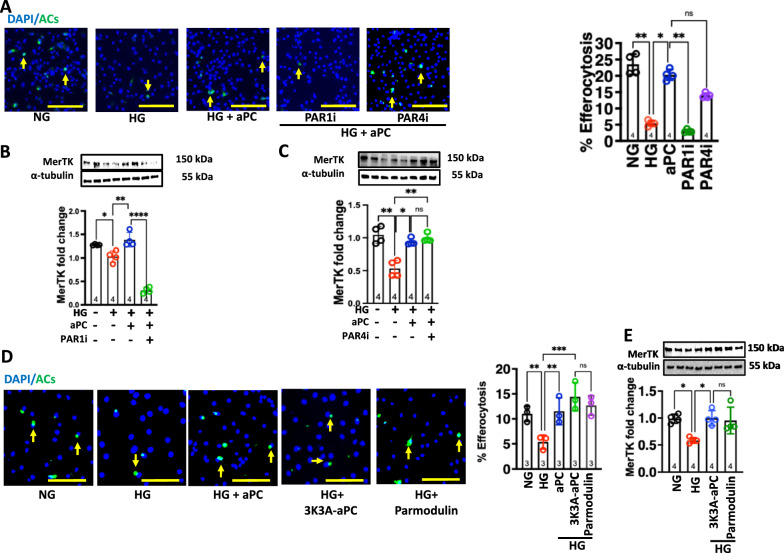
aPC maintains macrophage efferocytotic ability via protease activated receptor 1 (PAR1). **A–C** Representative co-immunofluorescence images showing efferocytotic ability (%) of the macrophages treated with PAR1i (P1Pal12, 10 µM) or PAR4i (P4Pal10, 5 µM), to engulf Vybrant DiO labelled apoptotic Jurkat cells (**A**, green). Yellow arrows indicate an association of ACs to macrophages (**A**, DAPI: blue, nuclear stain, scale bar represents 100 µm). Scatter plot with bar graphs summarizing results (**A**, right panel). Representative immunoblots showing expression levels of MerTK, α-tubulin: loading control. Scatter plot with bar graphs summarizing immunoblotting data for MerTK in PAR1i (**B**) and PAR4i (**C**) macrophages (N = 4). Data was analysed using one way ANOVA with Tukey post hoc comparison (B) and Kruskal Wallis test (**A, C**). **D, E** Representative co-immunofluorescence images showing efferocytotic ability of the macrophages (%) treated with aPC (20 nM), 3K3A-aPC (20 nM), or parmodulin-2 (20 nM) to engulf Vybrant DiO labelled apoptotic Jurkat cells (**D**, green). Yellow arrows indicate an association of ACs to macrophages (**D**, DAPI: blue, nuclear stain), scale bar represents 100 µm. Scatter plot with bar graphs summarizing results of efferocytosis in percentage (right panel). Representative immunoblots showing expression levels of MerTK, α-tubulin: loading control (**E**, upper panel) and scatter plot with bar graphs summarizing immunoblotting data for MerTK (**E**, lower panel) (D: N = 3, E: N = 4). Data was analysed using one way ANOVA with Tukey post hoc comparison (**D**) and Kruskal Wallis test (**E**). NG: normal glucose (5 mM glucose plus 20 mM mannitol), HG: high glucose (25 mM). HG + aPC: HG conditions with additional exposure to aPC (20 nM). HG + 3K3A-aPC: HG conditions with additional exposure to 3K3A-aPC (20 nM). HG + Parmodulin: HG conditions with additional exposure to parmodulin (20 nM). Each dot represents 1 biological sample. *P < 0.05, **P < 0.01, ***P < 0.001, ****P < 0.0001, ns; non-significant

To determine the functional relevance of these receptors for the effect of aPC on macrophage efferocytosis, we isolated BMDMs from wild-type mice and PAR1 or PAR4 function was blocked using specific inhibitory pepducins [15, 29]. These cells were exposed to NG or HG as described above. PAR1 inhibition efficiently abolished the effect of aPC on macrophage efferocytosis as reflected by aPC failure to induce ACs uptake by macrophages (Fig. 5A) and induction of MerTK levels (Fig. 5B), whereas PAR4 inhibition had no effect (Fig. 5A, C). Thus, PAR1 is required for the aPC-dependent maintenance of macrophage efferocytosis under high glucose conditions.

Considering that aPC functions both as an anticoagulant and cytoprotective agent [30], we evaluated the effect of 3K3A-aPC (variant lacking the anticoagulant function) and aPC signalling mimetic (parmodulin-2) on the efferocytotic ability of the macrophages exposed to HG. Both 3K3A-aPC and parmodulin-2 efficiently restored glucose impaired efferocytosis (Fig. 5D) and MerTK expression (Fig. 5E). Thus, mimicking aPC’s cytoprotective signalling is sufficient to restore efferocytosis in macrophages under high glucose conditions.

### aPC ameliorates diabetes-associated atherosclerosis via MerTK

**Fig. 6:**
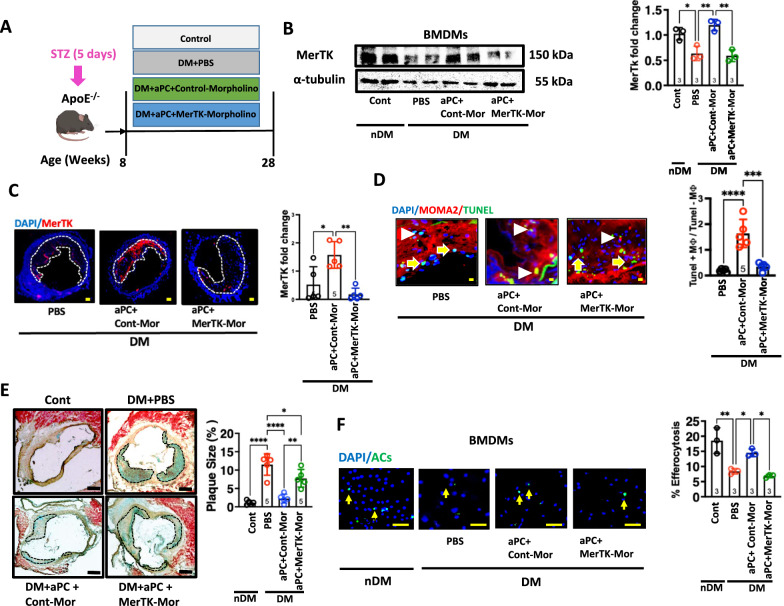
aPC ameliorates diabetes-associated atherosclerosis via MerTK. **A** Experimental scheme. **B** Representative immunoblots showing expression levels of MerTK, α-tubulin: loading control (left panel) and scatter plot with bar graphs summarizing immunoblotting data for MerTK (right panel) (N = 3). Data was analysed using one way ANOVA with Tukey post hoc comparison. **C** Representative co-immunofluorescence images of brachiocephalic artery showing expression of MerTK (red and DAPI: blue, nuclear stain, scale bar represents 50 µm, left panel) and scatter plot with bar graphs summarizing data (right panel) (N = 5). Data was analysed using one way ANOVA with Tukey post hoc comparison. **D** Representative images of brachiocephalic artery showing macrophage (MOMA-2, red) associated with tunnel positive apoptotic cells (green) assessed using in situ efferocytosis (left panel). White arrowheads indicate an association of macrophage to ACs. Scatter plot with bar graphs summarizing data (right panel). Scale bar represents 50 µm (N = 5). Data was analysed using one way ANOVA with Tukey post hoc comparison. **E** Representative images showing MOVATs staining of aortic roots (left panel). Scatter plot with bar graphs summarizing results obtained from MOVAT-staining (right panel). Scale bar represents 200 µm (N = 5). Data was analysed using one way ANOVA with Tukey post hoc comparison. **F** Representative co-immunofluorescence images showing efferocytotic ability of the macrophages (%) to engulf Vybrant DiO labelled apoptotic Jurkat cells (green). Yellow arrows indicate attachment of ACs to macrophages (DAPI: blue, nuclear stain), scale bar represents 100 µm. Scatter plot with bar graphs summarizing results of efferocytosis in percentage (right panel) (N = 3). Data was analysed using one way ANOVA with Tukey post hoc comparison. Cont: normoglycaemic control mice; DM + PBS: hyperglycaemic ApoE^−/−^ mice treated with PBS; DM + aPC + Cont-Mor: hyperglycaemic ApoE^−/−^ mice treated with control *vivo* morpholino and aPC; DM + aPC + MerTK-Mor: hyperglycaemic ApoE^−/−^ mice treated with MerTK-specific *vivo* morpholino and aPC. Each dot represents 1 biological sample. *P < 0.05, **P < 0.01, ***P < 0.001, ****P < 0.0001

We next determined whether aPC restores efferocytosis in macrophages via MerTK in vivo. We inhibited MerTK expression in diabetic ApoE^−/−^ mice using Vivo-Morpholinos (Fig. 6A). Diabetic ApoE^−/−^ mice (DM) with concomitant aPC treatment were injected with a MerTK specific morpholino (DM + aPC + MerTK-Mor) or a non-specific control morpholino (DM + aPC + Cont-Mor, Fig. 6A). Plasma lipids were comparable between the diabetic groups (Supplementary Fig. 4). The MerTK specific morpholino sufficiently reduced MerTK expression in the BMDMs and peritoneal macrophages (Fig. 6B and Supplementary Fig. 5), while a control morpholino had no impact. We next determined MerTK levels within atherosclerotic lesions. Increased levels of MerTK were observed within atherosclerotic lesions of diabetic mice treated with exogenous aPC plus control morpholino as compared to PBS treated diabetic mice (Fig. 6C). Conversely, decreased levels of MerTK were observed within atherosclerotic lesions of diabetic mice treated with exogenous aPC and MerTK specific morpholino (Fig. 6C). Of note, suppression of MerTK expression abolished the effect of aPC on macrophage efferocytosis (Fig. 6D and Supplementary Fig. 6A). Suppression of MerTK expression abolished the atheroprotective effect of aPC (Fig. 6E and Supplementary Fig. 6B, C). Next, we isolated BMDMs and peritoneal macrophages from the abovementioned mice and determined their efferocytotic ability ex vivo. Results revealed that exogenous aPC treatment prevented the diabetes-induced reduction in macrophages efferocytosis and that aPC’s effect was lost in diabetic ApoE^−/−^ mice treated with MerTK specific morpholino (Fig. 6F and Supplementary Fig. 7). Plasma levels of liver enzymes (ALT and AST) and blood urea nitrogen (BUN) were comparable between the diabetic groups treated with PBS or morpholions (Supplementary Fig. 8).

Collectively, these data indicate that the aPC-mediated amelioration of diabetes-associated atherosclerosis depends on MerTK-mediated macrophage efferocytosis.

## Discussion

While impaired TM-EPCR-mediated activation of protein C has been linked with diabetic complications, including atherosclerosis [11, 13, 14, 31, 32], its involvement in macrophage efferocytosis in diabetes-induced atherosclerosis remains unclear. Here we demonstrate that aPC restores glucose impaired macrophage efferocytosis in diabetes-induced atherosclerosis, using both human tissues and a murine model. Macrophages from diabetic patients exhibited reduced capacity to engulf apoptotic cells. aPC restored macrophage efferocytosis within atherosclerotic lesions and reduced plaque size in diabetic ApoE^−/−^ mice. However, when MerTK expression was inhibited in these mice, the protective effect of aPC was abolished, confirming that MerTK-mediated efferocytosis is essential for the aPC-mediated restoration of macrophage function. The current data suggest that inducing MerTK expression in macrophages by employing aPC-based therapies may be a feasible approach to restore macrophage efferocytosis capacity in diabetic conditions.

Our initial observations demonstrated that macrophages within the atherosclerotic lesions of diabetic patients exhibited impaired efferocytosis. Importantly, peripheral blood monocyte-derived macrophages of diabetic patients exhibited impaired efferocytosis. Ex vivo treatment with aPC efficiently restored efferocytosis in diabetic-conditioned hMDMs, highlighting the therapeutic potential of aPC in reversing the defective macrophage function associated with diabetes.

The restoration of efferocytosis by aPC was confirmed in vitro using BMDMs exposed to high glucose conditions. This effect was associated with increased MerTK expression and enhanced apoptotic cell clearance, which further suggests that aPC’s ability to restore efferocytosis is mediated through MerTK. The restoration of MerTK expression by aPC in diabetic macrophages is consistent with previous reports implicating MerTK as a key receptor for efferocytosis [6, 8–10]. Moreover, the ability of aPC to reverse the high glucose-induced downregulation of MerTK suggests that the protective effects of aPC on macrophage function are mediated, at least in part, through this receptor. The recent studies showing that targeted delivery of the MerTK protein in macrophages improves efferocytosis and reduces atherosclerosis in diabetic ApoE^−/−^ mice [8] and the aPC mediated restoration of MerTK in the current study supports the translational relevance of the current study.

Our study also indicates a role for Arg1 in the aPC-mediated restoration of macrophage efferocytosis. aPC treatment preserved Arg1 level in high glucose-exposed macrophages. These findings are consistent with previous reports showing that Arg1 promotes efferocytosis by converting arginine into pro-resolving metabolites such as ornithine [6]. Importantly, inhibition of Arg1 with the pharmacological inhibitor nor-NOHA abrogated the ability of aPC to maintain MerTK expression and macrophage efferocytosis under high glucose conditions, further implicating the importance of Arg1 for aPC mediated restoration of macrophage efferocytosis. These data are in agreement with previous studies, demonstrating that macrophages lacking Arg1 have defects in efferocytosis [6].

What might be the mechanisms underlying aPC-mediated restoration of glucose-impaired efferocytosis in macrophages? Here we show that aPC modulates the Rac1 levels and ATF6α cleavage, which are known to regulate macrophage efferocytosis, suggesting the regulation of efferocytosis by aPC involves Rac1 and ATF6α. In macrophages exposed to high glucose, we observed a decrease in Rac1, which is known to impair efferocytosis [33–35]. Treatment with aPC reversed this alteration, restoring the Rac1 activity and enhancing macrophage efferocytosis. This data is in agreement with previous studies showing that Rac1 promotes efferocytosis [25, 34, 35]. Furthermore, aPC treatment prevented the glucose-induced cleavage of ATF6α, which has been associated with atherosclerosis [12]. Contrary to our work, a previous study has shown that ATF6α induces MerTK and efferocytosis in CaMKIIγ-deficient macrophages under normal glucose conditions [36]. This discrepancy could be explained by a) different experimental conditions, e.g., high glucose-(current study) versus normal glucose (previous study), or b) differences in ATF6α detection method, in cleaved ATF6α (current study) or in total ATF6α (previous study) [36]. Inhibition of ATF6α cleavage by aPC is congruent with our previous work showing aPC-mediated ATF6α restriction in vascular endothelial cells [12] and reduced nuclear levels of ATF6α under high glucose conditions [37]. These data indicate that aPC exerts its protective effects on macrophage efferocytosis, at least in part, through regulation of Rac1-ATF6α signalling pathways. Yet, we cannot exclude a role of other signalling pathways in mediating aPC effect on macrophages efferocytosis.

Additionally, our study identifies PAR1 as a key receptor mediating aPC’s effects on macrophage efferocytosis. PAR1 is a key receptor through which aPC exerts its cytoprotective, anti-inflammatory, and pro-resolving effects in various cell types, including macrophages and endothelial cells [38, 39]. However, multiple co-receptors have been shown to complement aPC-PAR-1 signalling [30, 40, 41]. Thus, we cannot rule out the possibility of involvement of co-receptors for the aPC-PAR1 mediated restoration of glucose impaired macrophage efferocytosis.

Importantly, our experiments with 3K3A-aPC, (variant of aPC lacking anticoagulant activity and with parmodulin-2 (a small compound mimicking aPC’s PAR1 biased signaling) [42] showed that mimicking the signalling pathways of aPC could restore efferocytosis in macrophages. These findings suggest that selectively targeting aPC’s cytoprotective pathways, without its anticoagulant effects, could offer a more targeted and safer approach to treat diabetes-associated atherosclerosis. In vivo studies in diabetic ApoE^−/−^ mice confirmed that aPC treatment ameliorated the impaired efferocytosis and reduced atherosclerotic plaque size in diabetic animals. However, inhibition of MerTK expression abolished the vasculoprotective effects of aPC, further validating the importance of MerTK in mediating aPC’s effects on macrophage function and vasculoprotection. These results not only reinforce the in vitro and ex vivo findings but also provide strong evidence for the therapeutic potential of aPC’s cell signaling in preventing diabetes-associated atherosclerosis in vivo.

While providing significantly novel and useful insights, the current study has potential limitations. Only female mice were used in this study, which limits the applicability of the findings to both sexes. Due to ethical constraints and the exploratory nature of this study, we used n = 5 mice per group, larger cohorts are required to strengthen the study’s conclusion. The use of diabetic ApoE^−/−^ mice, while relevant, may not fully recapitulate the complex environment of human diabetes-associated atherosclerosis. Further studies in additional models are needed for broader applicability. Although MerTK was identified as a key mediator, the interplay of other efferocytosis-related receptors and pathways with aPC-mediated effects requires further exploration.

In summary, our findings demonstrate that aPC is valuable for maintaining macrophage efferocytosis in diabetes-induced atherosclerosis. The identification of MerTK, Arg1, and PAR1 as key mediators of aPC’s effects provides new insights into the mechanisms by which aPC regulates macrophage function. Our data suggest that enhancing macrophage efferocytosis by exogenous aPC treatment or by biased PAR1 agonists may provide promising therapeutic approaches for ameliorating diabetes-induced atherosclerosis.

## Supplementary Information

Final Supplemental File

## Data Availability

No datasets were generated or analysed during the current study.
