# Correction: Activated protein C ameliorates diabetes-induced atherosclerosis by sustaining macrophage efferocytosis

**DOI:** 10.1186/s12933-025-03058-z

**Published:** 2026-01-19

**Authors:** Saira Ambreen, Amna Arif, Saikal Shamkeeva, Ahmed Elwakiel, Surinder Pal, Shihai Jiang, Muhammad Asad Farhan, Zuhir Halloul, John H. Griffin, Berend Isermann, Khurrum Shahzad

**Affiliations:** 1 Institute of Laboratory Medicine, Clinical Chemistry and Molecular Diagnostics, Leipzig University, Paul-List-Straße 13/15, 04103 Leipzig, Germany; 2 Division of Vascular Surgery, Department of General, Abdominal and Vascular Surgery, Otto-Von-Guericke-University, Leipziger Straße 44, 39120 Magdeburg, Germany; 3 Department of Translational Medicine, The Scripps Research Institute, La Jolla, CA 92037 USA

**Correction to** **:** **Cardiovascular Diabetology (2025) 24:396**

10.1186/s12933-025-02965-5

In the original publication of this article [1], the author inadvertently missed the information in Acknowledgements, Author contributions, Funding and in the Ethical Approvals for animal experiments. The incorrect and correct versions are displayed below:

Acknowledgements

Incorrect version:

The authors thank Kathrin Deneser and Estela Mena Plaza for their technical support.

Correct version:

The authors thank Kathrin Deneser and Estela Mena Plaza for their technical support. Part of the in vivo work was done with the support of Azra Mehmood in the animal facility of the National Centre of Excellence in Molecular Biology, University of the Punjab, 87-West Canal Bank Road, Lahore, Pakistan.

Author contributions

Incorrect version:

S.A., performed the experiments, prepared the figures and prepared the first draft of the manuscript. A.A. performed in vitro experiment and conducted the manuscript revision work and S.S., performed the histological analyses. A. E., S.J., and M.A.F., assisted in histology. S. P. assisted in metabolomics data analysis. Z. H., provided human samples. J. G., provided reagents and assisted in manuscript preparation. B. I., interpreted the experimental work, critically reviewed data interpretation and assisted in manuscript preparation. K.S. conceptually designed and interpreted the experimental work and prepared the manuscript.

Correct version:

S.A., performed the experiments, prepared the figures and prepared the first draft of the manuscript. A.A. assisted in experiments and conducted the manuscript revision work and S.S., performed the histological analyses. A. E., S.J., and M.A.F., assisted in histology. S. P. assisted in metabolomics data analysis. Z. H., provided human samples. J. G., provided reagents and assisted in manuscript preparation. B. I., interpreted the experimental work, critically reviewed data interpretation and assisted in manuscript preparation. K.S. conceptually designed and interpreted the experimental work and prepared the manuscript.

Funding

Incorrect version:

Open Access funding enabled and organized by Projekt DEAL. This work was supported by grants from the Deutsche Forschungsgemeinschaft (SH849/4-1 to K. Shahzad; IS-67/25-1, IS-67/26-1 to B. Isermann), “Scientific Project Funding in the Field of Heart Medicine” at the Medical Faculty-University Hospital Leipzig to K. Shahzad, B. Isermann and from the US National Institutes of Health to J. H. Griffin (R01HL142975).

Correct version:

Open Access funding enabled and organized by Projekt DEAL. This work was supported by grants from the Deutsche Forschungsgemeinschaft (SH849/4-1 to K. Shahzad; IS-67/25-1, IS-67/26-1 to B. Isermann), “Scientific Project Funding in the Field of Heart Medicine” at the Medical Faculty-University Hospital Leipzig to K. Shahzad, B. Isermann, EFSD (European Foundation for the Study of Diabetes) to K. Shahzad, B. Isermann, and from the US National Institutes of Health to J. H. Griffin (R01HL142975).

Ethical Approvals for animal experiments

Incorrect version:

All animal experiments were conducted according to standards and procedures approved by the local Animal Care and Use Committee (Landesverwaltungsamt, Leipzig, Germany).

Correct version:

Mouse primary macrophages were isolated after confirmation of the related notification by the local Animal Care and Use Committee (Landesdirektion, Leipzig, Germany). In vivo animal experiments were conducted according to standards and procedures approved by the Institutional Animal Ethical Committee, National Centre of Excellence in Molecular Biology, University of the Punjab, Pakistan.

## References

[1] Ambreen S, Arif A, Shamkeeva S, et al. Activated protein C ameliorates diabetes-induced atherosclerosis by sustaining macrophage efferocytosis. Cardiovasc Diabetol. 2025;24:396. 41083981 10.1186/s12933-025-02965-5 PMC12519743

