# Supplementary material for: Activated protein C ameliorates diabetes-induced atherosclerosis by sustaining macrophage efferocytosis
Source: Cardiovasc Diabetol. 2025 Oct 13;24:396. doi: 10.1186/s12933-025-02965-5 (PMC12519743; doi:10.1186/s12933-025-02965-5)
Supplement: Supplementary file 1 — Final Supplemental File [file 12933_2025_2965_MOESM1_ESM.pdf]

Supplemental Information

Reagents

The following antibodies were used in the current study: rabbit anti-MerTK (AF591 Merck, R and D); rabbit anti-F4/80 (#MA1-91124, Thermofisher Scientific); rabbit anti-α-tubulin (Thermofisher Scientific), rat anti-MOMA-2 (#ab33451, Abcam), HRP-conjugated Affinipure Rabbit Anti-Goat IgG(H+L) (#SA00001-4, Proteintech), rabbit anti-mouse IgG HRP (#7076, Cell Signaling Technology). The following secondary antibodies for immunofluorescence were used: Goat anti-Rabbit IgG (H+L) Highly Cross-Adsorbed Secondary Antibody, Alexa Fluor Plus 647(A-11035, Invitrogen Goat Anti-Rabbit IgG (H+L), Superclonal™ Recombinant Secondary Antibody, Alexa Fluor 488 (#A27034, Invitrogen).

Other reagents were as follows: DMEM (#P04-03590, PAN BIOTECH), penicillin-streptomycin (#15140122, Thermo Fisher Scientific), FBS (#A5670701) and HEPES (#15630056, Gibco), RhoA/Rac1/Cdc42 activation assay combo kit (#STA-405, Cell Biolabs, Inc.), Albumin fraction (#8076.2, CARL ROTH), vectashield mounting medium with DAPI (#H-1200-10, Vector Laboratories), PVDF membrane and immobilon™ western chemiluminescent HRP substrate (Merck, Millipore, United States), streptozotocin (Enzo Life Sciences, Germany), Oil Red-O (# 00625, Sigma-Aldrich, Germany), L929 cell line (Sigma-Aldrich, Germany), accu-chek test strips, accu-check glucometer, protease inhibitor cocktail (Roche Diagnostics, Germany), hematoxylin Gill II, acrylamide (Carl ROTH, Germany), aqueous mounting medium (ZYTOMED, Germany), "high fat diet" (HFD) experimental food (Western Type Diet, 43% carbohydrates, 15% proteins and 42%, Ssniff, Germany), ketamine 10% (beta-pharm, Germany).

Major Resources Table

Animals (in vivo studies)

Species	Vendor or Source	Background Strain	Sex	Persistent ID / URL
Mus musculus	Janvier Labs	C57BL/6J Mouse	Female	https://www.janvier-labs.com/en/fiche_produit/c57bl-6jrj_mouse/
Mus musculus	Jackson Labs	ApoE$^{-/-}$	Female	https://www.jax.org/search?q=Apoe-/-

Antibodies

Target antigen	Vendor or Source	Catalog #	Working concentration	Persistent ID / URL

Phospho-CaMKII (Thr286) (D21E4)	Cell Signaling Technology	12716	1:1000	https://www.cellsignal.com/products/primary-antibodies/phospho-camkii-thr286-d21e4-rabbit-mab/12716
Mouse Mer Antibody	R and D	AF591	1:1000	https://www.rndsystems.com/products/mouse-mer-antibody_af591
Human Mer Antibody	R and D	AF891	1:1000	https://www.rndsystems.com/products/human-mer-antibody_af891
F4/80 Monoclonal Antibody	Thermo fisher Scientific	MA1-91124	1:1000	https://www.thermofisher.com/antibody/product/F4-80-Antibody-/MA1-91124
Rac1/cdc42	Cell Signaling Technology	4651	1:1000	https://www.cellsignal.com/products/primary-antibodies/rac1-cdc42-antibody/4651
Thrombomodulin (1009) Mouse Monoclonal Antibody	Merck Millipore	339M-1	1:100	https://www.sigmaaldrich.com/DE/de/product/sigma/339m1
ATF6-alpha	Santa Cruz	22799	1:1000	https://datasheets.scbt.com/sc-22799.pdf
EPCR	Proteintech	67658-1-Ig	1:100	https://www.ptglab.com/de/products/PROCR-Antibody-67658-1-Ig.htm
alpha-tubulin	Cell Signaling Technology	2144	1:1000	https://www.cellsignal.com/products/primary-antibodies/a-tubulin-antibody/2144
Anti-Monocyte + Macrophage antibody [MOMA-2]	Abcam	ab33451	1:50	https://www.abcam.com/monocyte--macrophage-antibody-moma-2-ab33451.html
PAR1 Antibody	Novus Biologicals	NBP1-71770	1:1000	https://www.novusbio.com/products/par1-thrombin-receptor-antibody-n2-11_nbp1-71770
PAR4 Antibody	Boster	A03645-1	1:1000	https://www.bosterbio.com/anti-par-4-f2rl3-antibody-a03645-1-boster.html
Rabbit anti-GAPDH	Sigma Aldrich	G9545	1:10000	https://www.sigmaaldrich.com/catalog/product/sigma/g9545?lang=en&region=CA
Anti-mouse IgG, HRP-linked Antibody	Cell Signaling Technology	7076	1:4000	https://www.cellsignal.com/products/secondary-antibodies/anti-mouse-igg-hrp-linked-antibody/7076?site-search-type=Products

Anti-rabbit IgG, HRP-linked Antibody	Cell Signaling Technology	7074	1:4000	https://www.cellsignal.com/products/secondary-antibodies/anti-rabbit-igg-hrp-linked-antibody/7074?site-search-type=Products
HRP-conjugated Affinipure Rabbit Anti-Goat IgG(H+L)	Proteintech	SA00001-4	1:4000	https://www.ptglab.com/products/HRP-conjugated-Affinipure-Rabbit-Anti-Goat-IgG-H-L-secondary-antibody.htm
Goat anti-Rabbit IgG (H+L) Highly Cross-Adsorbed Secondary Antibody, Alexa Fluor Plus 647	Invitrogen	A-11035	1:300	https://www.thermofisher.com/antibody/product/Goat-anti-Rabbit-IgG-H-L-Highly-Cross-Adsorbed-Secondary-Antibody-Polyclonal/A- A32733
Goat anti-Rabbit IgG (H+L), Superclonal™ Recombinant Secondary Antibody, Alexa Fluor 488	Invitrogen	A27034	1:300	https://www.thermofisher.com/antibody/product/Goat-anti-Rabbit-IgG-H-L-Secondary-Antibody-Recombinant-Polyclonal/A27034
3K3A-aPC	Dr. John H. Griffin, Department of Molecular Medicine, The Scripps Research Institute, La Jolla, CA, USA			

Reagents and Kits

Reagents	Vendor or Source	Catalog #	Persistent ID / URL
Recombinant Human MCSF	Peprotech	300-25	https://www.peprotech.com/en/recombinant-human-m-csf
Lymphoprep ™	Stem Cell Biotechnologies	07861/ 07851	https://www.stemcell.com/products/lymphoprep.html
Nω-Hydroxy-nor-L-arginine, Diacetate Salt - CAS 189302-40-7 - Calbiochem	Merck	399275	https://www.merckmillipore.com/DE/de/product/N-Hydroxy-nor-L-arginine-Diacetate-Salt-CAS-189302-40-7-Calbiochem,EMD_BIO-399275?ReferrerURL=https%3A%2F%2Fwww.google.com%2F
ON-TARGETplus siRNA Mouse MerTK	Dharmacon	L-040357-00-005	https://horizondiscovery.com/en/gene-modulation/knockdown/sirna/products/on-targetplus-sirna-reagents
PAR1-Pal12	Gene Script		
PAR4-Pal10	Gene Script		
ML161 (Parmodulin 2)	STELLCHEM	S2916	https://www.selleckchem.com/datasheet/ml-161-S291601-DataSheet.html
RPMI 1640	PAN BIOTECH	P04-16500	https://shop.pan-biotech.de/RPMI-1640-w-L-Glutamine-w-2.0-g-L-NaHCO3/P04-16500
DMEM	PAN BIOTECH	P04-03590	https://shop.pan-biotech.de/en/DMEM-w-4.5-g-L-Glucose-w-L-Glutamine-w-Sodium-pyruvate-w-3.7-g-L-NaHCO3/P04-03590
FBS	Gibco	A5670701	https://www.thermofisher.com/order/catalog/product/de/de/A5670701
Hematoxylin solution acc. to Gill II	CARL ROTH	T864.2	https://www.carlroth.com/com/en/cytological-routine-staining/hematoxylin-solution-acc-to-gill-ii/p/t864.2
Agarose Standard	CARL ROTH	3810.3	https://www.carlroth.com/com/en/agarose-gelling-reagents/agarose-standard/p/3810.3
Aqueous Mounting Medium	ZYTOMED	ZY-AMT030	https://www.zytomed-systems.com/storage/uploads/datasheets/en/ZY-AMT_EN.pdf
In situ cell death detection kit, fluorescence	Roche	11684795910	In Situ Cell Death Detection Kit, Fluorescein sufficient for ≤50 tests, suitable for detection | Sigma-Aldrich (sigmaaldrich.com)
Pierce™ BCA protein assay kit	Perbio Science	23227	https://www.thermofisher.com/order/catalog/product/23227#/23227
Vector shield mounting	Vector Laboratories	H-1200-10	https://vectorlabs.com/vectashieldr-plus-antifade-mounting-medium-with-dapi.html

medium with DAPI			
PVDF membrane	Merck Millipore	IPVH00010	https://www.merckmillipore.com/DE/de/search/IPVH00010?search=&TrackingSearchType=SB+-+homepage-search-box+-+OLD&SearchContextPageletUUID=&SearchTerm=IPVH00010
Nitrocellulose/Filter Paper Sandwich, 0.2 µm, 8.3 x 7.3 cm	Invitrogen™	LC2000	Nitrocellulose/Filter Paper Sandwich, 0.2 µm, 8.3 x 7.3 cm (thermofisher.com)
Immobilion™ chemiluminescent HRP substrate	Merck Millipore	WBKLS0500	https://www.merckmillipore.com/DE/de/search/WBKLS0500?search=&TrackingSearchType=SB+-+Search+Result+Search+Box&SearchContextPageletUUID=&SearchTerm=WBKLS0500
Powdered milk	CARL ROTH	T145.2	https://www.carlroth.com/de/de/blockierungsreagenzien/milchpulver/p/t145.2
Albumin fraction	CARL ROTH	8076.2	https://www.carlroth.com/de/de/blockierungsreagenzien/albumin-fraktion-v/p/8076.2
Rotiphorese® Gel	CARL ROTH	3029.1	https://www.carlroth.com/de/de/page-fertigloesungen/rotiphoresegel-30-%28375%3A1%29/p/3029.1
Donkey serum	Sigma Aldrich	D9663	https://www.sigmaaldrich.com/catalog/product/sigma/d9663?lang=en&region=CA
Phosphate Buffer Saline (PBS)	Thermo Fisher Scientific	10-010-031	https://www.thermofisher.com/order/catalog/product/10010031?SID=srch-hj-10010031#/10010031?SID=srch-hj-10010031
Rompun 2%	Bayer		https://covetrus.de/a/rompun-2prozent-flasche-25-ml/02.2049.25256.770081
Ketamine	Beta-pharm		
Tween-20	CARL ROTH	9127.1	https://www.carlroth.com/de/de/reagenzien-zur-dna-rna-analyse/tween-20/p/9127.1
2-Propanol	CARL ROTH	CP41.1	https://www.carlroth.com/de/de/von-a-bis-z/2-propanol/p/cp41.1
Eosin Y Solution, Alcoholic, with Phloxine	Sigma Aldrich	HT110316	https://www.sigmaaldrich.com/DE/de/product/sigma/ht110316
MOVAT's Pentachrome Staining	Morphisto	12057	https://www.morphisto.de/en/shop/detail/d/F%C3%A4rbekit%3A_MOVAT_Pentachrom_%28Original%29/9150/
Oil Red O	Sigma Aldrich	O0625	https://www.capitolscientific.com/Sigma-Aldrich-O0625-25G-Oil-Red-O-Certified-Biological-Stain-25g-Bottle
Natriumchlorid, 5 kg, Kunst.	CARL ROTH	9265.2	https://www.carlroth.com/de/de/von-a-bis-z/natriumchlorid/p/9265.2

Dodecylsulfate·Na-salt	Serva	20760.02	https://www.serva.de/enDE/ProductDetails/369_20760_Dodecylsulfate_Na_salt_cryst_research_grade.html
Paraformaldehyde	Sigma-Aldrich	818715	https://www.merckmillipore.com/DE/de/product/Paraformaldehyde,MDA_CHEM-818715?ReferrerURL=https%3A%2F%2Fwww.google.com%2F
TRIS Hydrochlorid	CARL ROTH	9090.1	TRIS Hydrochlorid, 50 g, CAS No. 1185-53-1 | Reagenzien für die Proteinisolierung | Proteinaufreinigung | Proteinbiologie | Life Science | Carl Roth - Deutschland
HBSS	Thermo Fisher Scientific	14025-050	https://www.thermofisher.com/order/catalog/product/14025100#/14025100
Protease Inhibitor Cocktail	Roche Diagnostics	11 836 153 001	https://www.sigmaaldrich.com/catalog/product/roche/11836153001?lang=en&region=CA
HEPES	Gibco	15630056	https://www.fishersci.de/shop/products/hepes-1m-4/11560496
Penicillin-Streptomycin	Thermo Fisher Scientific	15140122	https://www.thermofisher.com/order/catalog/product/15140122

Analysis of blood lipids

Blood lipids (total cholesterol, LDL-cholesterol and triglyceride) were measured with the Cobas 111® c analyzer from Roche Diagnostics (Basel, Switzerland).

Histology and Immunohistochemistry

MOVAT's staining was performed on frozen sections of aortic roots. Frozen sections (5 µm) were fixed in Bouin's solution at 50°C for 10 min and stained with 5% sodium thiosulfate for 5 min, 1% alcian blue for 15 min, alkaline alcohol for 10 min, Movat's Weigert's solution for 20 min, crocein scarlet acid / fuchsin solution for 1 min, 5 % phosphotungstic acid for 5 min and 1% acetic acid for 5 min. Between every staining step the tissue sections were washed with tap water and distilled water. Afterwards, they were dehydrated in 95% and 100% ethanol for 1 min and stained in alcohol saffron for 8 min. Tissue sections were washed in 100% ethanol for 1 min, moved to Xylol for 10 min and covered with cytoseal mounting medium. For histological analysis images were captured with an Olympus Bx43-Microscope (Olympus, Hamburg, Germany). Image J software was used for image analysis

For immunofluorescence, frozen sections of truncus brachiocephalic arteries with maximum plaque size were fixed in ice cold acetone for 8 min, washed twice with ice cold PBS and incubated in 2% BSA in PBST for 1 h. Sections were then incubated for overnight at 4°C with one or two primary antibodies against MerTK, or MOMA-2. Sections incubated without

primary antibodies were used as negative controls for background correction. After overnight incubation the sections were washed three times with 1 x PBS five min each time followed by incubation with fluorescently labelled corresponding secondary antibodies. After washing, nuclear counterstaining was conducted using mounting medium with DAPI. Images were visualized, captured, and analysed using a fluorescence microscope. All histological analyses were performed by two independent blinded investigators. Immunofluorescence images were captured with an Olympus Bx43-Microscope (Olympus, Hamburg, Germany). The Image J software was used for image analysis.

Human atherosclerotic carotid plaque sections were washed in 1x PBS and were then incubated with a 3% peroxidase block (30 % hydrogen peroxide in 1x PBS) for 10 min followed by a 1h incubation in the blocking solution. Sections were then incubated for one night at 4 °C with a primary antibody against TM and EPCR (1:100 each). Washed with 1x PBS and then HRP secondary added for 30 min and washed again 3 times. After that DAB substrate was added for 6 min. Washed with PBS and mounted using mounting media and then analysed under the microscope.

Isolation and culture of bone marrow derived macrophages

Bone marrow derived macrophages (BMDMs) were isolated and cultured as described previously [1]. Briefly, 10 to 12 weeks old C57BL/6J mice were sacrificed by cervical dislocation and bones were isolated from hind limbs (tibia, femur). Bones were kept in and flushed with RPMI-1640 complete medium to isolate bone marrow cells. Bone marrow cells were further washed with 1 x PBS and resuspended in culture medium RPMI-1640 supplemented with 30% L929 cell-conditioned medium (supernatant collected from fibroblast L929 cells, cultured for 10 days in DMEM growth medium, supplemented with 10% FBS and 1% penicillin–streptomycin) and 20% FBS. This procedure was repeated twice to remove dead cells. After the final washing step pelleted cells were resuspended in above culture medium. Cells were cultured for 7 to 10 days until ~80% confluence. The purity of cells was confirmed by F4/80 staining using FACS analyses and was consistently found to be higher than 90%. These cells were used as BMDM for experiments. To knock down expression of MerTK, cells were transfected with either scrambled siRNA control and oligo-targeting MerTK (siMerTK, 50 nM) using Turbofect (Life Technologies) reagents following the manufacturer’s instructions. To inhibits Arginase 1, cells were treated with Arginase 1 inhibitor (Nor-NOHA:500mM) followed by co-culturing with labelled apoptotic Jurkat cells. PAR-1 or PAR4-signalling was blocked

using specific pepducins peptides, (PAR1; P1pal-12 peptide RSLSSSAVANRS, 10nM and PAR4; P4pal-12 peptide RSLSSSAVANRS, 5uM) 30min before the efferocytosis assay.

Analyses of human samples

Diabetes was diagnosed in patients according to the American Diabetes Association criteria. Diabetic (N=6) and non-diabetic (N=8) patients with atherosclerotic disease were recruited from the cardiology clinic at the University Hospital Magdeburg. All 6 diabetic patients were type 2 diabetic patients (T2DM). All patients and controls were Caucasian. All patients were newly admitted to the university hospital at the Otto-von-Guericke University, Magdeburg, for treatment of ACI-stenosis, which was henceforth the primary diagnosis in all patients. Detailed information about the patient's clinical characteristics is given in supplementary table S1. Tissue biopsies of atherosclerotic plaques were obtained from internal carotid artery during carotid disobliteration. Samples were immediately embedded in OCT compound and snap frozen. Tissue biopsies were sectioned at 6 µm thickness and used for immunofluorescence staining as described above. The study complied with the Declaration of Helsinki and all patients entered the study according to the guidelines of the local ethics committees after giving informed consent (Ethic-Committee-No: 92/09). Not all endpoints were measured in each donor due to sample unavailability issues.

Immunoblotting

Proteins were isolated and immunoblotting was performed as described [1 2 3]. Cell lysates were prepared in RIPA buffer (50 mM Tris at pH 7.4, 1% Nonidet P-40, 0.25% sodium deoxycholate, 150 mM NaCl, 1 mM EDTA, 1 mM Na_3VO_4, and 1 mM NaF supplemented with protease inhibitor cocktail). Lysates were centrifuged (10.000 × g, 10 min at 4°C) and insoluble debris was discarded. The protein concentration in supernatants was quantified using BCA reagent. Equal amounts of protein were electrophoretically separated on 7.5%, 10% (vol/vol), or 12.5% (vol/vol) SDS polyacrylamide gels, transferred to PVDF membranes, and probed with the desired primary antibodies overnight at 4°C. Membranes were then washed with PBS-tween (PBST) and incubated with anti-mouse, anti-rat IgG, or anti-rabbit IgG (each 1: 2,000) horseradish peroxidase-conjugated antibodies, as indicated. Blots were developed with the enhanced chemiluminescence system. To compare and quantify levels of proteins, the density of each band was measured using ImageJ. Equal loading was confirmed by immunoblotting with α-tubulin antibody.

Rac1 activation assay

Rac1 activation assays was performed using PAK-PBD affinity beads, according to the manufacturer's instructions (Cell Biolabs, Inc.). Briefly, BMDMs cultures were exposed to normal glucose (5mM) or high glucose (25mM) without or with aPC (20 nM), for the desired period. The growth medium was removed, and the cells were rinsed twice with ice-cold PBS and lysed in ice-cold cell lysis buffer (provided in the kit). The cell lysates were centrifugation at 14,000 × g at 4°C for 10 min. The supernatant was collected and protein concentration in each sample was determined. Equal concentrations of protein (700 µg) were incubated overnight at 4°C with PAK-PBD beads (40µl each/sample). The lysates were pelleted by centrifugation at 14000 × g, for 10 sec and the supernatant was aspirated. The pellet was washed 3x with 1x washing buffer assay. PAK-PBD bead pellet was resuspended in 40 µl of 2× Laemmli buffer and boiled for 5 min and subjected to centrifugation (14000 × g, for 10 sec). The active Rac1-GTP and total Rac1 in each sample were assessed by immunobloting using anti-Rac1 antibody, respectively.

Assessment of plasma levels of liver enzymes and blood urea nitrogen

Plasma levels of liver enzymes (Alanine transaminase, ALT and Asparate transaminase, AST) and blood urea nitrogen (BUN) were determined using Cobas 111® c analyzer (Roche Diagnostics).

Preparation of activated protein C

Activated protein C was generated as previously described [1]. Prothrombin complex (Prothromplex NF600), containing all vitamin K dependent coagulation factors, was reconstituted with sterile water and supplemented with $CaCl_2$ at a final concentration of 20 mM. The column for purification of protein C was equilibrated at RT with 1 liter of washing buffer (0.1 M NaCl, 20 mM Tris, pH 7.5, 5 mM benzamidine HCl, 2 mM Ca^{2+}, 0.02% sodium azide). The reconstituted prothombin complex was gravity eluted on a column filled with Affigel-10 resin covalently linked to a calcium-dependent monoclonal antibody to PC (HPC4). The column was washed first with two column volumes of washing buffer and then two column volumes with wash buffer rich in salt (0.5 M NaCl, 20 mM Tris, pH 7.5, mM benzamidine HCl, 2 mM Ca^{2+}, 0.02% sodium azide). Then the benzamidine was washed off the column with a buffer of 0.1 M NaCl, 20 mM Tris, pH 7.5, 2 mM Ca^{2+}, 0.02% sodium azide. To elute PC, the column was gravity eluted with elution buffer (0.1 M NaCl, 20 mMTris, pH 7.5, 5 mM EDTA, 0.02% sodium azide, pH 7.5) and 3 ml fractions were collected. The peak fractions were

identified by measuring absorbance at 280 nm. The peak fractions were pooled. The recovered PC was activated with human plasma thrombin (5% w/w, 3 h at 37°C). To isolate activated protein C (aPC) ion exchange chromatography with FPLC (ÄKTAFPLC®, GE Healthcare Life Sciences) was used. First, thrombin was removed with a cation exchange column MonoS (GE Healthcare Life Sciences). Then a MonoQ anion exchange column (GE Healthcare Life Sciences) was equilibrated with 10% of a 20 mM Tris, pH 7.5, 1 M NaCl buffer. After applying the solution that contains aPC a 10-100% gradient of a 20 mM Tris, pH 7.5, 1 M NaCl buffer was run through the column to elute aPC at a flow of 1-2 ml/min under continuous monitoring of OD and conductivity. aPC eluted at ~36 mS/cm by conductivity or at 40% of the buffer. Fractions of 0.5 ml were collected during the peak and pooled. Proteolytic activity of purified aPC was ascertained with the chromogenic substrate SPECTROZYME® PCa.

Isolation of peripheral blood monocytes and generation of human monocyte-derived macrophages (hMDMs)

We used the Lymphoprep™ (catalogue # 07861/ 07851 Stem Cell Biotechnologies) to isolate human peripheral blood mononuclear cells (PBMCs) from healthy and diabetic adult volunteers (Ethic Committee No: 281/22-ek) according to the protocol. Then, PBMCs were treated with differentiated medium (RPMI-1640, 10% HI-FBS, 50ng/mL hM-CSF (human macrophage colony-stimulating factor 1) for 10 days to differentiate them into macrophages (hMDMs). After 10 days of proliferation and differentiation, hMDMs obtained were used for experiments.

***In situ* efferocytosis assay**

Frozen sections were immersed in OCT and cryo-sectioned, and 5-µm sections were permeabilized in ice-cold acetone for 20 min. Sections were incubated with TUNEL staining reagents at 37°C for 60 min and then washed three times with PBS. Sections were then first blocked for 60 min in blocking buffer (BCA) at room temperature, then incubated overnight at 4°C with the following antibodies: anti-MOMA-2 or anti-F4/80 antibody. Sections were washed with PBS and incubated with corresponding fluorescently-labelled secondary antibodies and counterstained with DAPI. Images were captured using a Keyence microscope. Quantification was done by counting TUNEL positive cells, associated with MOMA-2+ macrophages versus macrophage-free TUNEL poistive cells. Macrophage-associated apoptotic cells were TUNEL positive nuclei in contact with or in close proximity with neighbouring MOMA-2+ macrophages. Free apoptotic cells were not in contact with neighbouring macrophages.

Supplementary table S1: Clinical characteristics of patients with or without diabetes mellitus.

**Parameter**	**Group statistics**		***P*-value**
	**Non-DM** (N=8)	**DM** (N=6)	Non-DM vs DM
**Age (years)**	63.39 ± 1.214	62.17 ± 1.341	NS
**Sex (M/F)**	6/2	4/2	NS
**Diabetes duration (years)**	-	15.33 ± 1.142	-
**HbA1c (%)**	4.88 ± 0.053	7.65 ± 0.234	<0.01
**CAD (Y/N)**	4/4	4/2	NS
**RAS (Y/N)**	2/6	2/4	NS
**HTN (Y/N)**	4/4	2/4	NS
**Chol (mmol/L)**	6.51 ± 0.012	3.56 ± 0.190	0.02
**LDL (mmol/L)**	4.90 ± 0.571	2.41 ± 0.176	<0.01
**BMI (kg/m^2)**	32.91 ± 1.096	30.88 ± 0.7462	NS
**Smoking (Y/N)**	2/6	2/4	NS
**Oral antidiabetic medication (Y/N)**	-	5/1	-
**Insulin treatment (Y/N)**	-	3/3	-
**Lipid lowering drug (Y/N)**	4/4	1/5	<0.01
**antihypertensive drug (Y/N)**	4/4	2/4	NS
**Platelet activation inhibitors (Y/N)**	3/5	2/4	NS

Supplementary Table 1. Clinical characteristics of patients from which plaque biopsies were obtained. Data were obtained at the time of biopsy. Abbreviations: M: Male, F: Female, BMI: body mass index, Y: Yes, N: No, ACI: internal carotid artery, HTN: Hypertension, CAD: Coronary Artery Disease, RAS: Renal Artery Stenosis, Chol: Cholesterol. For statistical analysis of Sex the Fisher's exact test was used and data are shown as mean ± SEM. For all other parameters analyses were performed using the unpaired two-tailed student's *t*-test. For significant differences the *P*-values are shown (NS: not significant).

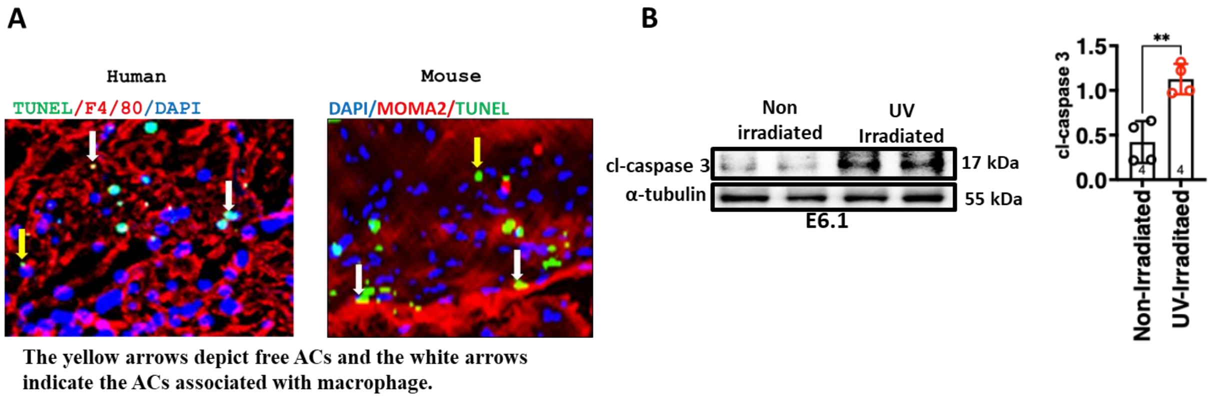

Supplementary Figure 1. Assessment of cl-caspase 3 in UV irradiated Jurkat cells.

A: Representative immunofluorescence images showing in situ efferocytosis assessed by counting TUNEL+ nuclei that were associated with Mac2+ macrophages ("associated"), indicative of efferocytosis, or not associated with macrophages ("free").

B: Representative immunoblots showing expression levels of cleaved (cl) caspase-3, α-tubulin: loading control (left panel) and scatter plot with bar graphs summarizing immunoblotting data for cleaved caspase-3 (right panel). (N=4) Data was analysed using Unpaired t test,**P<0.01.

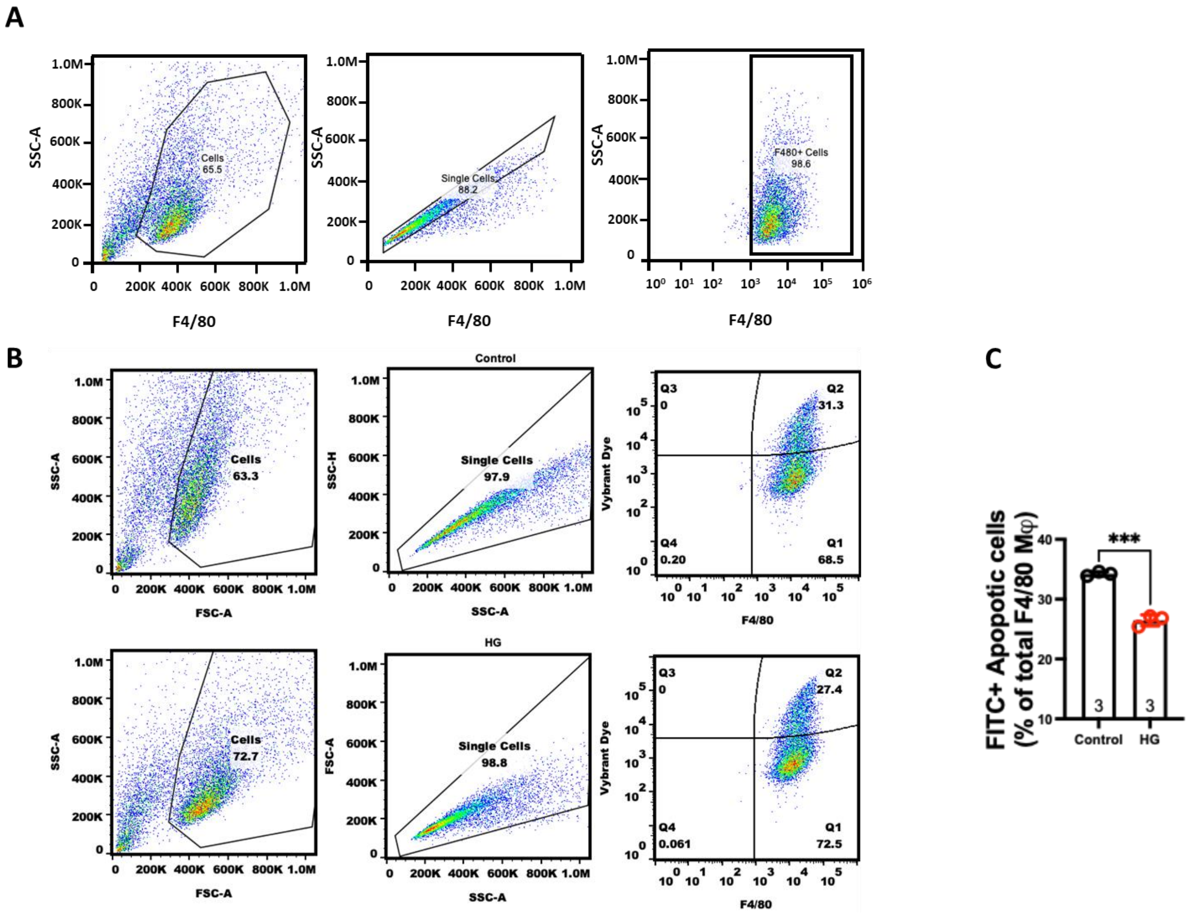

Supplementary Figure 2. Assessment of efferocytosis in BMDMs by flow cytometry.

A: Gating strategy for the FACS analyses of bone marrow derived macrophages. BMDMs were harvested and analysed by flow cytometry. FSC-A/SSC-A gating to identify cells of interest (left panel). SSC-H and SSC-A gating to identify single cells (middle panel). SSC-A and F4/80 gating to identify macrophages (right panel).

B, C: Gating strategy for the FACS analyses of efferocytosis. Scatter plot with bar graphs summarizing macrophage efferocytosis data analyzed by flow cytometry. (N=3) Data was analysed using Unpaired t test, ***P<0.001.

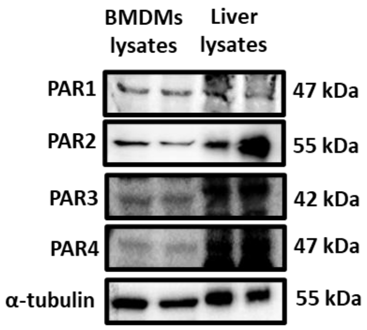

Supplementary Figure 3. Assessment of presence of PARs in macrophages.

Representative immunoblots showing expression levels of PAR1-PAR4, α-tubulin: loading control in BMDMs, liver was used as positive control.

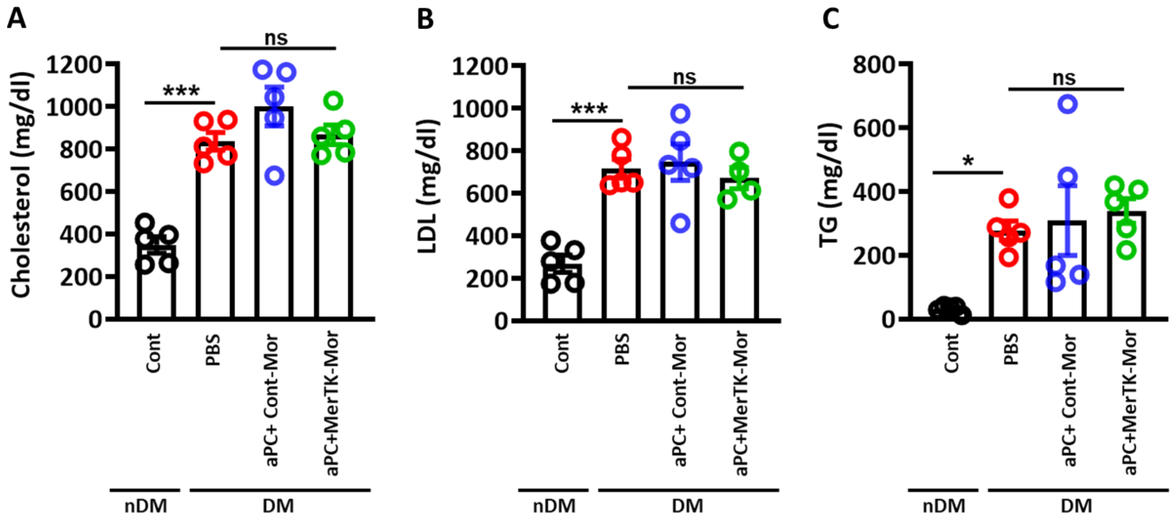

Supplementary Figure 4: Assessment of plasma lipid levels.

Plasma total cholesterol (A), low density lipoprotein, LDL (B) and triglyceride, TG (C) levels in DM+PBS: hyperglycaemic ApoE-/- mice treated with PBS; DM+aPC+Cont-Mor: hyperglycaemic ApoE-/- mice treated with control vivo morpholino and aPC; DM+aPC+MerTK-Mor: hyperglycaemic ApoE-/- mice treated with MerTK-specific vivo morpholino and aPC. (N=4-5) Data was analysed by using one way ANOVA with Tukey post-hoc comparison. *P<0.05, ***P<0.001, ns non-significant.

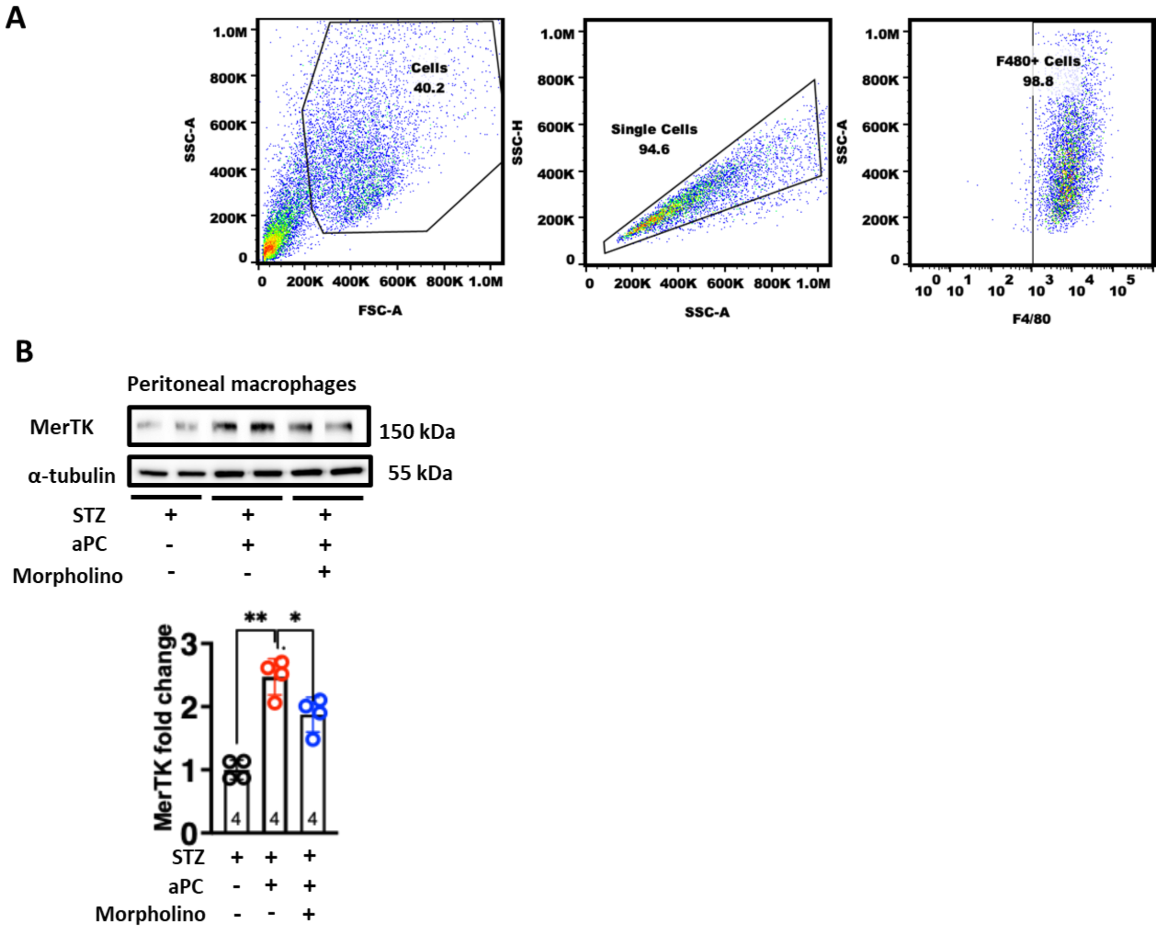

Supplementary Figure 5. Assessment of MerTK expression and efferocytosis in peritoneal macrophages by flow cytometry.

A: Gating strategy for the FACS analyses of peritoneal macrophages. peritoneal macrophages were harvested and analysed by flow cytometry. FSC-A/SSC-A gating to identify cells of interest (left panel). SSC-H and SSC-A gating to identify single cells (middle panel). SSC-A and F4/80 gating to identify macrophages (right panel).

B: Representative immunoblots showing expression levels of MerTK, α-tubulin: loading control (top panel) and scatter plot with bar graphs summarizing immunoblotting data for MerTK (bottom panel). (N=4) Data was analysed using Kruskal Wallis test. *P<0.05, **P<0.01.

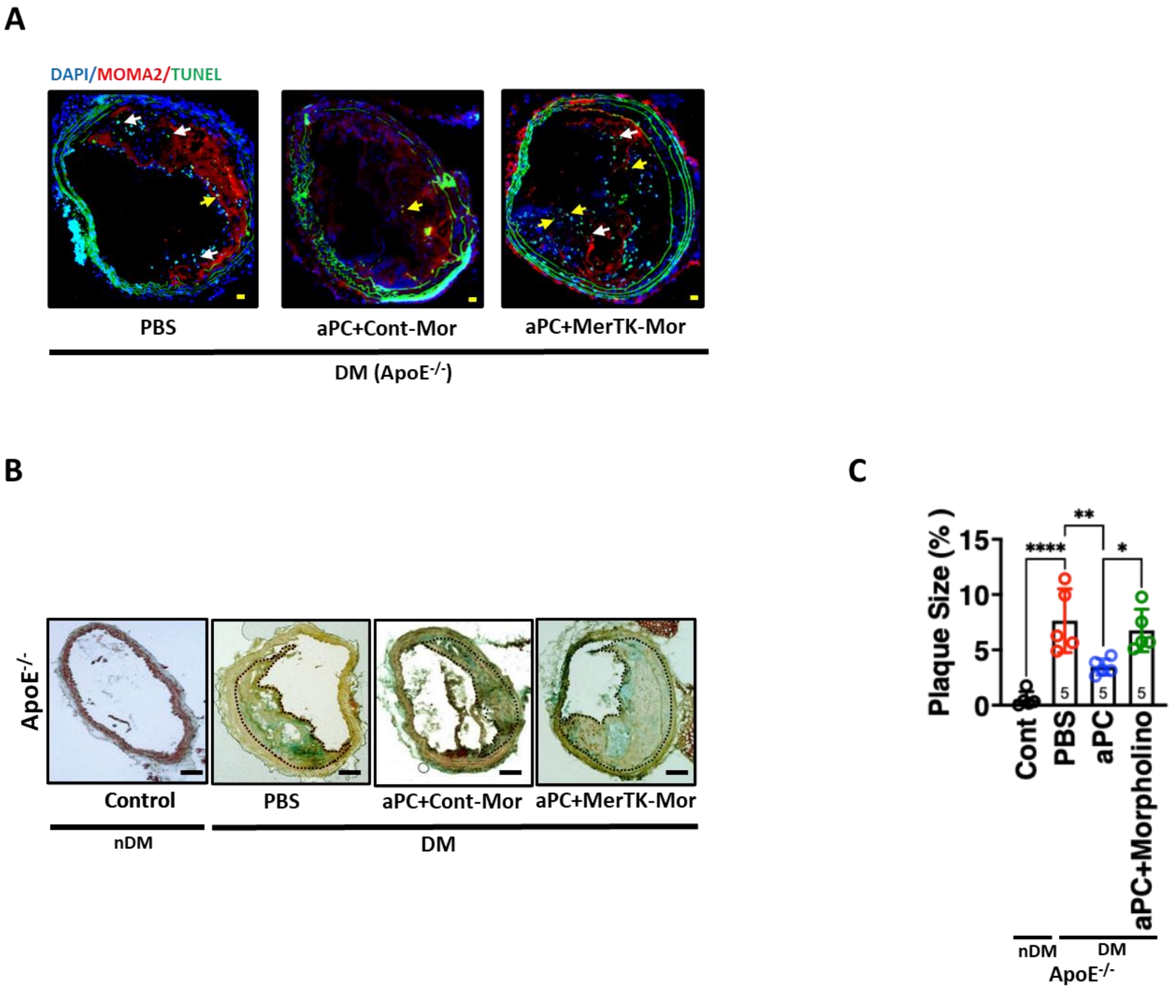

Supplementary Figure 6. aPC ameliorates diabetes-associated plaque formation via MerTK in truncus brachiocephalic artery

A: Representative images of brachiocephalic artery showing macrophage (MOMA-2, red) associated with TUNEL positive apoptotic cells (green) assessed using *in situ* efferocytosis. White arrows indicate an association of macrophage to ACs (right panel). Scale bar represents 50µm.

B, C: Representative images showing MOVATs staining of truncus brachiocephalic artery (B). Scatter plot with bar graphs summarizing results obtained from MOVAT-staining (C). Scale bar represents 200µm). Data was analysed using Kruskal Wallis test.

Cont: normoglycaemic control mice; DM+aPC+Cont-Mor: hyperglycaemic ApoE$^{-/-}$ mice treated with control *vivo* morpholino and aPC; DM+aPC+MerTK-Mor: hyperglycaemic ApoE$^{-/-}$ mice treated with MerTK-specific *vivo* morpholino and aPC. (N=5) Data was analysed one way ANOVA with Tukey post-hoc comparison. *P<0.05, **P<0.01 ****P<0.0001.

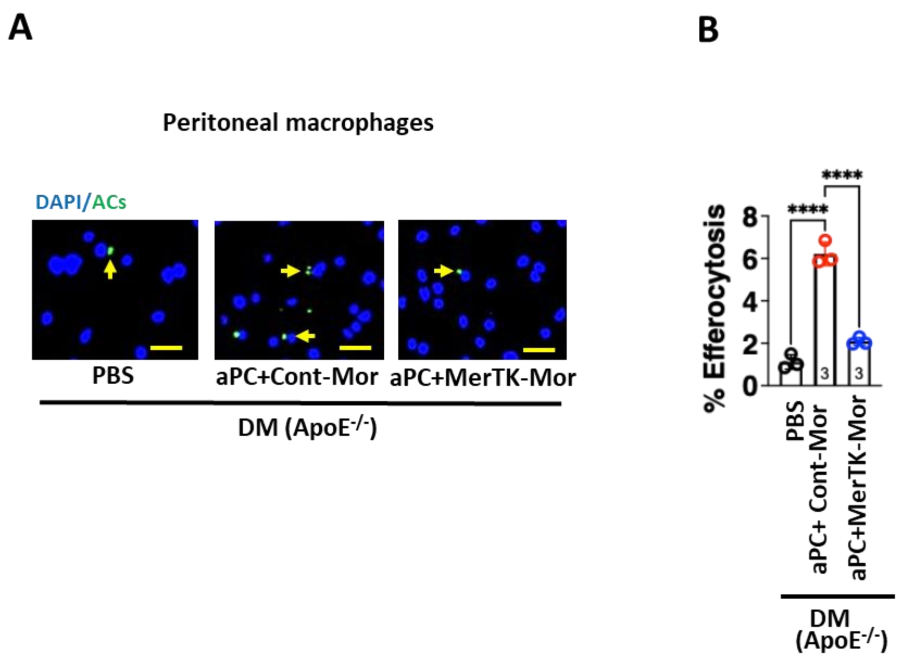

Supplementary Figure 7. aPC maintains macrophage efferocytotic ability via MerTK

A, B: Representative co-immunofluorescence images showing efferocytotic ability of the peritoneal macrophages to engulf Vybrant DiO labelled apoptotic Jurkat cells (green, **A**). Yellow arrows indicate an association of apoptotic cells to macrophages (DAPI: blue, nuclear stain), scale bar represents 100µm. Scatter plot with bar graphs summarizing results of efferocytosis in percentage (**B**).

DM+PBS: hyperglycaemic ApoE$^{-/-}$ mice treated with PBS; DM+aPC+Cont-Mor: hyperglycaemic ApoE$^{-/-}$ mice treated with control *vivo* morpholino and aPC; DM+aPC+MerTK-Mor: hyperglycaemic ApoE$^{-/-}$ mice treated with MerTK-specific *vivo* morpholino and aPC. Each dot represents 1 biological sample (N=3). Data was analysed by one way ANOVA with Tukey post-hoc comparison. ****P<0.0001.

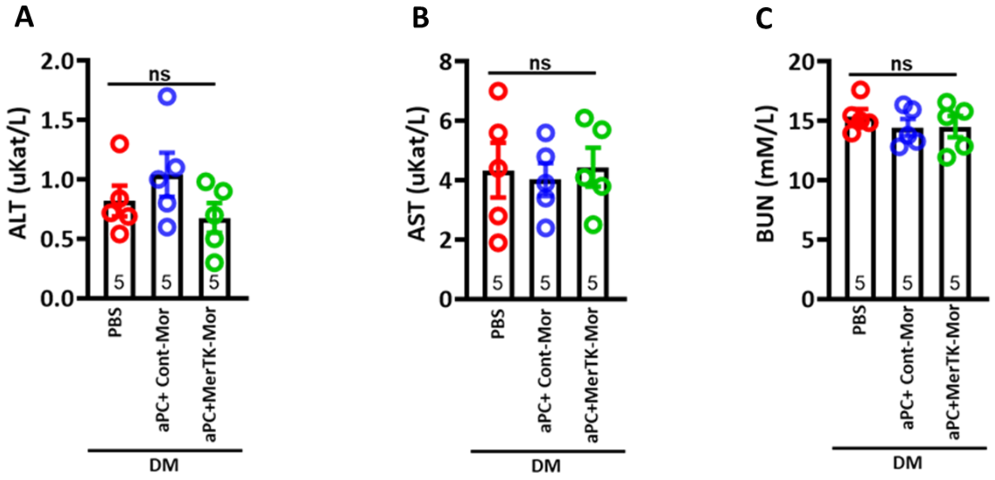

Supplementary Figure 8: Assessment of plasma ALT, AST and BUN lipid levels.

A-C: Plasma levels of liver enzymes and blood urea nitrogen. Plasma ALT (A), AST (B) and BUN (C) levels.

DM+PBS: hyperglycaemic ApoE-/- mice treated with PBS; DM+aPC+Cont-Mor: hyperglycaemic ApoE-/- mice treated with control vivo morpholino and aPC; DM+aPC+MerTK-Mor: hyperglycaemic ApoE-/- mice treated with MerTK-specific vivo morpholino and aPC. Each dot represents 1 biological sample (N=5). Data was analysed by one way ANOVA with Tukey post-hoc comparison. ns, non-significant.

References:

1. Shahzad, K. *et al.* Activated protein C reverses epigenetically sustained p66Shc expression in plaque-associated macrophages in diabetes. *Commun. Biol.* **1**, 104 (2018).
2. Elwakiel, A. *et al.* Factor XII signaling via uPAR-integrin β1 axis promotes tubular senescence in diabetic kidney disease. *Nat. Commun.* **15**, 7963 (2024).
3. Manoharan, J. *et al.* Tissue factor binds to and inhibits interferon-α receptor 1 signaling. *Immunity* **57**, 68-85.e11 (2024).
